# The national and provincial disease and indirect economic burden of high alcohol use in China from 1990 to 2023 with forecasting to 2050

**DOI:** 10.1016/j.mmr.2026.100043

**Published:** 2026-06-05

**Authors:** Yang Yang, Da-Chuang Zhou, Zhen-Ping Zhao, Ke-Jia Zhou, Di Zhang, Yu-Ting Xia, Peng Yin, Mai-Geng Zhou, Wen-Xi Tang

**Affiliations:** a National Center for Chronic and Noncommunicable Disease Control and Prevention, Chinese Center for Disease Control and Prevention, Beijing 100050, China; b Center for Pharmacoeconomics and Outcomes Research & Department of Public Affairs Management, School of International Pharmaceutical Business, China Pharmaceutical University, Nanjing 211198, China; c School of International Pharmaceutical Business, China Pharmaceutical University, Nanjing 211198, China

**Keywords:** High alcohol use, Disease burden, Indirect economic burden, Spatiotemporal analysis, Forecasting, China

## Abstract

**Background:**

High alcohol use, defined as consumption exceeding the theoretical minimum risk exposure level, poses a major public health challenge in China, yet comprehensive long-term estimates combining disease burden and indirect economic burdens are limited. This study quantified national and subnational trends in disease and indirect economic burdens attributable to high alcohol use in China from 1990 to 2023 and forecasted the high alcohol use-related indirect economic burden to 2050.

**Methods:**

Disease burden data were obtained from the Global Burden of Disease Study 2023. Economic parameters were sourced from the China Statistical Yearbook 2024, the Penn World Table, and the Seventh National Population Census. The indirect economic burden was estimated using a cost-of-illness model. The estimated annual percentage change (EAPC) was calculated to assess trends (1990−2023) in age-standardized mortality and DALY rates, as well as productivity losses due to premature death, morbidity, and total indirect economic burden. Spatial distributions of all-cause burden and the top six leading diseases (ranked by DALYs) were compared between 1990 and 2023. Sex and age-specific patterns were analyzed using absolute DALY numbers and indirect economic burden values. Finally, the Bayesian age-period-cohort model was used to forecast productivity losses due to premature death and morbidity, along with the total indirect economic burden to 2050.

**Results:**

National age-standardized mortality rate attributable to high alcohol use declined from 27.68/100,000 [95% uncertainty interval (UI) 13.17−48.69] in 1990 to 10.58/100,000 (95% UI 4.60−19.26) in 2023 (EAPC=−3.02%, 95% UI −4.15 to −1.85; *P*<0.01), and the age-standardized DALY rate attributable to high alcohol use declined from 1012.53/100,000 (95% UI 567.90−1565.46) to 492.91/100,000 (95% UI 311.38−751.27) (EAPC=−2.26%, 95% UI −3.05 to −1.52; *P*<0.01). Corresponding indirect economic burden as a percentage of Gross Domestic Product (GDP) decreased from 0.15% (95% UI 0.13−0.18) to 0.13% (95% UI 0.11−0.16) (EAPC=−0.47%, 95% UI −0.77 to −0.17; *P*<0.01). High disease burden consistently clustered in Southwest and North China, while high economic burden mainly concentrated in East and Southwest, with notable regional mismatches. Burden was higher in males than in females, and the age profile of indirect economic loss shifted toward older working-age adults. Forecasts indicated that the overall indirect economic burden as a percentage of GDP reached a nadir around 2039 (EAPC=0.12%, 95% UI 0.10–0.14), before rising to 0.14% (95% UI 0.12–0.21) in 2050.

**Conclusions:**

From 1990 to 2023, age-standardized mortality and DALY rates attributable to high alcohol use declined substantially in China, and the indirect economic burden as a share of GDP decreased modestly, despite marked growth in absolute productivity loss. Subnational heterogeneity persisted among different regions. Forecasts of the indirect economic burden indicate a nadir around 2039, followed by a subsequent increase with uncertainty.

## Background

As a psychoactive and toxic substance with dependence-producing properties, alcohol has long been recognized as a major health hazard; most recently, the 2024 International Agency for Research on Cancer Handbook on Cancer Prevention reconfirmed the causal role of alcoholic beverage consumption in at least seven cancer types [1]. According to the 2024 Global Status Report on alcohol and health and treatment of substance use disorders, alcohol use accounted for 2.60 million deaths globally in 2019, 4.70% of all deaths, with about 2.00 million among males [2]. Robust epidemiological evidence links alcohol use to a wide spectrum of diseases and injuries, and global comparative assessments within the Global Burden of Disease (GBD) framework have systematically quantified these harms across cancers, liver cirrhosis, cardiovascular diseases, and road injuries [3]. In the current GBD risk taxonomy, high alcohol use denotes consumption above the theoretical minimum risk exposure level (TMREL) [4]. GBD 2016 supported a near-zero all-age TMREL [3], while GBD 2020 introduced age-varying TMREL [5]. Consistent with these updates, alcohol use remains a top-10 contributor to all-age disability-adjusted life years (DALYs) globally [4].

Beyond health loss, the economic loss is substantial. The latest global systematic review and modelling study estimates alcohol-attributable costs at 2.6% [95% confidence interval (CI) 2.0−3.1] of global Gross Domestic Product (GDP) after adjustment for omitted components, with indirect costs (primarily productivity loss from premature deaths, absenteeism and presenteeism) accounting for 61.2% (95% CI 55.0−71.4) of total costs [6]; earlier synthesis works likewise found indirect costs to be the largest share in most settings [6], [7]. In China, a recent provincial analysis estimated that, in 2020, the total societal cost attributable to modifiable risk factors in Shanghai was US$7.9 billion [95% uncertainty interval (UI) 4.6–12.4], driven largely by productivity losses of US$5.4 billion (95% UI 3.2–8.3). Alcohol use accounted for US$1.1 billion (95% UI 0.7–1.6), representing 13.3% (95% UI 8.2–19.7) of the total attributable societal cost [8].

In this context, the Chinese government has enacted a series of public-health measures, including the criminalization of drunk driving in 2011 [9], [10], tightened advertising regulations in 2015 [11], and explicit alcohol-reduction targets in the “Healthy China 2030” strategy [12]. Given China’s vast territory and pronounced regional disparities, comprehensive estimation and forecasting of the disease burden and indirect economic burden attributable to high alcohol use at both regional and national levels remain limited.

To address these gaps, we quantified the disease burden of high alcohol use using data from the GBD study. Additionally, we constructed a cost-of-illness (COI) model to estimate the indirect economic burden of high alcohol use as a percentage of GDP in China for 1990 and 2023. We further analyzed temporal, geographic, sex, and age differences in both disease burden and indirect economic burden. Finally, we applied a Bayesian age-period-cohort (BAPC) model to estimate the future disease burden in China due to high alcohol use from 2024 to 2050. We then calculated the indirect economic burden, providing comprehensive evidence to inform policy prioritization. Overall, the purpose of this study was to quantify and project the disease burden and indirect economic burden attributable to high alcohol use in China across time, region, sex, and age, thereby providing evidence to support the prioritization and tailoring of alcohol-control policies and resource allocation.

## Methods

### Data sources

The GBD 2023 Study synthesized global incidence, prevalence, and probability of death data to produce systematic, up-to-date estimates of fatal and non-fatal health loss for 375 diseases and injuries across 204 countries and territories from 1990 to 2023 [13], [14], [15], [16]. Data on deaths, DALYs, and years lived with disability (YLDs) attributable to high alcohol use, including all causes and the six leading Level-3 causes of DALYs, were obtained for China (1990–2023) from the GBD Study database, along with probability of death and population data [17]. Provincial and national GDP and per capita GDP figures for China from 1990 to 2023 were sourced from the China Statistical Yearbook 2024 [18]. The proportion of labor income in GDP was derived from the Penn World Table database [19], while labor force participation rates were obtained from the Seventh National Population Census of China [20]. GDP and probability of death from 2024 to 2050 for forecasting indirect economic burden were also sourced from the GBD studies [21], [22]. The classification criteria for regions adopted in this study were consistent with those reported in previously published literature [23], [24]. Details of the data extracted from GBD 2023, together with a summary of data sources, key parameters, and assumptions used for estimating indirect economic burden, are listed in **Additional file 1:** Tables S1-S2.

### Disease burden of high alcohol use

We quantified the national and provincial disease burden attributable to high alcohol use by extracting data on deaths, DALYs, and YLDs for China in 1990 and 2023 from the GBD database [17]. Estimates for cause-specific deaths were derived via the cause of death ensemble model and harmonized with all-cause mortality using the cause of death correction algorithm; conversely, non-fatal outcomes were quantified using Disease Modeling Meta-Regression version 2.1 (DisMod-MR 2.1) [18], [20]. The analysis describes the spatial distribution of burden attributable to high alcohol use. It includes all-cause burden and the burden attributable to the top six Level-3 causes of alcohol-attributable DALYs across all ages. These causes were: alcohol use disorders, stroke, esophageal cancer, cirrhosis and other chronic liver diseases, falls, and liver cancer [14], [16].

GBD studies estimate high alcohol use by integrating multi-source data, which is adjusted to account for tourist and unrecorded alcohol consumption, and Spatiotemporal Gaussian Process Regression (ST-GPR) modeling is used to generate exposure estimates for each region, year, age, and sex [16]. Relative risk (RR) is curved for specific diseases, which is derived from systematic literature reviews of cohort and case-control studies, and meta-regression-Bayesian regularized, trimmed (MR-BRT) methods are used [16]. The TMREL is calculated independently for each region, age, sex, and year by weighting the RR curve of each relevant cause of death by its share of DALYs in that population and then taking the minimum point of the resulting overall risk curve [16]. Finally, the population attributable fraction (PAF) is calculated by comparing the population’s actual risk with a counterfactual scenario, in which all consumption above the TMREL is reduced to the TMREL, thereby quantifying the disease burden caused by high alcohol use [16]. The GBD world population age structure served as the reference for standardizing rates, and uncertainty was expressed as 95% UIs, derived from the 2.5th and 97.5th percentiles of 1000 draws [14], [15], [16].

To characterize subnational variations across China, covering both all-cause burdens and the top six specific causes, we utilized the all-age DALY rate (defined as total DALYs divided by the total population). We selected this crude rate because it preserves the actual age structure of each region, thereby reflecting the disease burden under current demographic conditions and aligning more closely with real-world healthcare demand and macroeconomic pressure. Furthermore, we assessed sex and age disparities in all-cause DALYs through descriptive analyses of model-based estimates.

### Estimation of indirect economic burden

We applied the cost-of-illness approach to estimate the corresponding indirect economic burden and its percentage of GDP. Comparative analyses were conducted to examine differences in burden over time, by province, disease, sex, and age. Parameters used in this section and the reasons for their selection are listed in the **Additional file 1:** Table S2.

Based on the COI approach, this study employed the morbidity costs and premature death costs to estimate the indirect economic burden attributable to high alcohol use [24], [25]. As shown in Formula (1), the indirect economic burden is defined as the productivity loss of morbidity and premature deaths caused by risk factors. This burden could be further divided into productivity loss of premature deaths ($\mathrm{IC}D_{a_{l}}$), estimated on the basis of the number of individuals exiting the labor market early, and productivity loss of morbidity ($\mathrm{IC}Y_{a_{l}}$), referring to loss of high alcohol use-related diseases, primarily quantified using YLDs. We applied two indicators to assess the relative economic impact of the indirect economic burden. The primary indicator was indirect economic burden as a percentage of GDP. The secondary indicator was indirect economic burden as one ten-thousandth of GDP, which allows a more precise graphical presentation of the indirect economic burden attributable to a single disease.

(1) $$IC_{\mathrm{al}}=\mathrm{IC}Y_{\mathrm{al}}+\mathrm{IC}D_{\mathrm{al}}$$

(2) $$\mathrm{IC}Y_{\mathrm{al}}=\left(\alpha G_{L}L_{\mathrm{al}}+\alpha {\lambda \mu G}_{L}\;\left(1-L_{\mathrm{al}}\right)\right)\times \;\text{YLD}\mathrm{al}$$

The variables used in the model include regional per capita GDP ($G_{L}$), age-specific labor force participation rates ($L_{\mathrm{al}}$), the proportion of labor income in GDP (α), the value of household productivity (λ), and the income conversion ratio for household production (μ). Because researches show that unemployed individuals may engage in household production [26], [27], the primary analysis in this study assumed a specified level of household productivity, with the income conversion ratio for household production set at 50.00% and a wide-ranging one-way uncertainty analysis (±50%) as well as 1000 Monte Carlo simulations incorporating nearly all parameters are performed to estimate indirect economic burden to address parameter uncertainty [28]. A summary of the parameter settings, sources, and rationale is provided in **Additional file 1:** Table S2. As shown in Formula (2), after calculating the income level for each age group, multiply it by the corresponding YLDs, the product is $\mathrm{IC}Y_{\mathrm{al}}$.

As detailed in Formula (3), the indirect economic burden from premature death was calculated by projecting the lifetime income lost for everyone who died prematurely due to high alcohol use. This was achieved by using provincial life tables to determine the probability of an individual surviving to each future age, assuming no premature death had occurred [15]. This survival probability was then multiplied by the projected income for each corresponding future age to calculate the total expected lifetime income. To account for the time value of money, these future income losses were discounted to their present value. Based on established economic parameters, we assumed an annual income growth rate of 5.00% and applied a discount rate of 5.00% [18], [29]. For losses occurring in 1990 and 2023, the values were discounted to their respective years to ensure comparability. This discounted lifetime income value was then multiplied by the number of premature deaths attributable to high alcohol use, as reported in the GBD database, to determine the total indirect economic cost of premature death.

(3) $$\;\mathrm{IC}D_{\mathrm{al}}=\sum_{t=1}^{100}\left[\pi_{\mathrm{al},t}\left(\alpha G_{L}L_{\mathrm{al}}+\alpha {\lambda \mu G}_{L}\;\left(1-L_{\mathrm{al}}\right)\right){\left(\frac{1+g}{1+r}\right)}^{t-1}\right]\times \;\text{Death}\mathrm{al}$$

(4) $$\;{\text{π}}_{a_{l},t}=\prod_{k=0}^{t-1}\left(1-q_{a+k,l}\right)$$

Within this calculation, $\pi_{\mathrm{al},t}\;$denotes the probability, derived from provincial life tables, of an individual in age group *a* surviving to each future year *t* (up to 100 years of age) $q_{a+k,l}\;$[as shown in Formula (4)] [28]. $q_{a+k,l}\;$denotes the probability of death for individuals ageda+k in location l. *g* is the assumed annual wage growth rate, set at 5.00%, and *r* is the annual discount rate, set at 5.00% [18], [29].

We assessed uncertainty in the estimated indirect economic burden using deterministic one-way uncertainty analyses and probabilistic uncertainty analyses. For the one-way uncertainty analyses, each input parameter was varied individually to its prespecified lower and upper bounds while holding all other parameters constant, and the resulting estimates were recorded. For probabilistic uncertainty analysis, we conducted 1000 Monte Carlo simulations; in each iteration, parameters were sampled from their respective uncertainty distributions (**Additional file 1:** Table S2) and the indirect economic burden was recalculated. We reported 95% UIs, defined as the 2.5th and 97.5th percentiles of the 1000 simulated estimates [30].

### .1 Trend analysis

We calculated the estimated annual percentage change (EAPC) to quantify the temporal trends in the burden attributable to high alcohol use from 1990 to 2023 with a log-linear regression model as listed in Formula (5) and (6).

(5) $$\;\log\left(\mathrm{rat}e_{t}\right)=\alpha +\beta t+\varepsilon_{t}$$

(6) $$\text{EAPC}=100\times \left(\left[\exp\left(\text{β}\right)-1\right)\right]$$

Where ${\text{rate}}_{\text{t}}$ denotes the age-standardized rate in year *t*, *t* is the calendar year, α is the intercept, β is the regression coefficient for the time trend, and $\varepsilon_{t}$ is the error term.

Random draws of 1000 samples were obtained from the distributions of age-standardized rates for DALY and death, productivity loss due to death and morbidity (% of GDP), indirect economic burden (% of GDP), and an EAPC was calculated for each draw. The 95% UIs were defined as the 2.5th and 97.5th percentiles of the ordered EAPC estimates, providing a robust measure of variability. Final EAPC estimates were the mean values across 1000 draws. To assess the statistical evidence for temporal trends, we derived a two-sided tail probability from the simulated distribution of the regression coefficient *β* by calculating the proportions of *β*>0 and *β<*0, and taking twice the smaller proportion. For comparisons between the 1990 and 2023 estimates of indirect economic burden (% of GDP), we computed draw-level paired differences and calculated two-sided *P*-values using paired t-tests; Wilcoxon signed-rank tests were performed as a non-parametric sensitivity analysis.

### Forecasting the indirect economic burden

To forecast the indirect economic burden attributable to high alcohol use, we applied a BAPC modelling framework implemented using integrated nested Laplace approximation [31]. This approach was used to project deaths and YLDs attributable to high alcohol use from 2024 to 2050. To ensure model robustness, data from 1990−2015 was used for model training, and data from 2016−2023 was retained for out-of-sample validation. Predictive performance was assessed by comparing model-based estimates with observed values using the root-mean-square error (RMSE) and the mean absolute percentage error (MAPE). UIs for RMSE and MAPE were obtained using nonparametric bootstrap resampling. We resampled the evaluation dataset with replacement (1000 replicates), recalculated RMSE and MAPE for each resample, and defined the 95% UI as the 2.5th and 97.5th percentiles of the empirical bootstrap distribution [32].To assess the future indirect economic burden associated with high alcohol consumption, we drew on GDP projections from a published study that modelled China’s future economic growth using multisource datasets [17], [33]. These models centered on the log-increment of GDP among the working-age population and incorporated structural covariates, lagged growth components, and autoregressive residual terms to characterize long-run economic trends and stochastic variability. We forecasted productivity loss due to deaths (% of GDP), productivity loss due to morbidity (% of GDP), and the total indirect economic burden (% of GDP) from 2024 to 2050, and reported age-specific forecasts for 2030, 2040, and 2050.

### Statistical analysis

Uncertainty was quantified using 95% UIs, derived from GBD draws, Monte Carlo simulations, or bootstrap resampling, as appropriate. Statistical analyses, modeling, forecasting, Monte Carlo simulations, and visualizations were performed using R software (version 4.4.2). Where hypothesis testing was performed, all tests were two-sided and P < 0.05 was considered statistically significant.

## Results

### Trend analysis

At the national level, the all-cause ASMR attributable to high alcohol use was 27.68/100,000 (95% UI 13.17−48.69) in 1990 and 10.58/100,000 (95% UI 4.60−19.26) in 2023 (Table 1). Similarly, the age-standardized DALY rate was 1012.53/100,000 (95% UI 567.90–1565.46) in 1990 and 492.91/100,000 (95% UI 311.38–751.27) in 2023 (Table 1). Regarding the indirect economic burden, the overall indirect economic burden as a percentage of GDP was 0.15% (95% UI 0.13−0.18) in 1990 and 0.13% (95% UI 0.11−0.16) in 2023 (Table 1).

**Table 1:** High alcohol use attributable all causes ASMR, age-standardized DALY rate, and indirect economic burden in China.

**Parameter**	**1990 (95% UI)**			**2023 (95% UI)**		
	**Disease burden (/100,000)**		**Indirect economic burden (% of GDP)**	**Disease burden (/100,000)**		**Indirect economic burden (% of GDP)**
	**ASMR**	**Age-standardized DALY rate**		**ASMR**	**Age-standardized DALY rate**	
**China**	27.68 (13.17−48.69)	1012.53 (567.90−1565.46)	0.15 (0.13−0.18)	10.58 (4.60−19.26)	492.91 (311.38−751.27)	0.13 (0.11−0.16)
**Northeast**						
Heilongjiang	33.21 (12.92−64.26)	1137.41 (552.31−1921.29)	0.12 (0.10−0.14)	11.31 (4.17−22.76)	514.33 (296.72−838.06)	0.12 (0.09−0.13)
Jilin	32.85 (15.19−59.25)	1119.18 (635.95−1827.31)	0.14 (0.12−0.17)	9.83 (4.39−18.35)	466.46 (302.02−706.14)	0.12 (0.09−0.13)
Liaoning	26.49 (10.50−50.00)	892.13 (451.43−1454.58)	0.13 (0.10−0.15)	11.57 (4.36−21.77)	482.95 (278.58−748.18)	0.11 (0.09−0.13)
**North**						
Beijing	20.50 (7.98−38.12)	721.19 (414.23−1115.79)	0.13 (0.11−0.15)	6.77 (2.94−11.93)	365.40 (251.17−524.57)	0.11 (0.09−0.13)
Hebei	25.59 (10.47−45.51)	854.41 (440.93−1363.26)	0.12 (0.10−0.14)	12.87 (5.16−23.81)	480.11 (258.03−758.27)	0.11 (0.09−0.12)
Inner Mongolia	31.44 (11.24−59.57)	1201.67 (646.87−1855.07)	0.19 (0.15−0.21)	11.14 (3.59−21.19)	615.28 (379.08−941.03)	0.17 (0.14−0.19)
Shanxi	23.85 (10.28−42.09)	883.01 (502.06−1366.42)	0.13 (0.11−0.16)	8.74 (3.71−16.41)	421.53 (275.11−631.36)	0.11 (0.09−0.13)
Tianjin	20.91 (7.03−42.12)	714.97 (353.35−1186.19)	0.11 (0.09−0.13)	8.16 (3.02−14.99)	379.81 (253.41−566.77)	0.09 (0.07−0.10)
**East**						
Anhui	29.02 (12.38−51.07)	1081.60 (585.24−1658.16)	0.15 (0.12−0.18)	9.77 (3.96−18.48)	459.49 (288.87−685.43)	0.12 (0.10 − 0.15)
Fujian	29.32 (12.50−52.34)	1151.21 (621.51−1829.27)	0.17 (0.14−0.20)	10.47 (4.22−18.73)	533.39 (344.90−769.17)	0.16 (0.14−0.19)
Jiangsu	19.92 (8.51−36.76)	752.53 (420.15−1162.99)	0.14 (0.12−0.17)	10.07 (4.16−18.72)	417.47 (252.88−634.66)	0.11 (0.09−0.13)
Jiangxi	27.67 (14.24−46.28)	1018.05 (621.34−1521.56)	0.15 (0.12−0.18)	10.35 (4.68−18.35)	464.19 (295.33−686.97)	0.13 (0.11−0.15)
Shandong	24.63 (9.61−43.93)	865.03 (456.79−1363.83)	0.14 (0.11−0.16)	9.32 (3.07−17.35)	421.10 (249.54−642.21)	0.11 (0.09−0.13)
Shanghai	17.36 (6.96−31.54)	670.82 (389.48−1004.54)	0.15 (0.13−0.18)	5.72 (2.79−9.70)	344.97 (245.05−465.43)	0.12 (0.10−0.14)
Zhejiang	26.75 (12.09−47.10)	944.70 (533.47−1446.51)	0.17 (0.14−0.20)	9.93 (4.81−17.54)	425.79 (285.69−605.33)	0.14 (0.12−0.16)
**South**						
Guangdong	24.94 (10.89−44.62)	1010.98 (563.16−1567.88)	0.16 (0.13−0.19)	8.45 (3.63−15.78)	465.48 (303.25−700.89)	0.15 (0.12−0.17)
Guangxi	30.09 (15.62−49.46)	1104.39 (672.92−1667.37)	0.14 (0.11−0.17)	12.08 (5.59−21.38)	537.90 (337.30−809.09)	0.12 (0.10−0.15)
Hainan	19.81 (10.33−31.37)	791.36 (491.46−1157.09)	0.11 (0.09−0.14)	7.93 (3.80−13.81)	403.87 (269.11−584.02)	0.11 (0.09−0.13)
**Central**						
Henan	29.20 (11.35−55.78)	1041.00 (506.55−1756.56)	0.14 (0.11−0.17)	10.79 (3.81−21.16)	512.18 (304.42−767.26)	0.13 (0.11−0.15)
Hubei	36.83 (17.77−65.37)	1218.84 (685.14−1952.12)	0.17 (0.14−0.20)	9.59 (3.10−18.28)	462.12 (275.58−694.51)	0.14 (0.12−0.16)
Hunan	28.50 (15.26−47.04)	985.46 (604.52−1467.69)	0.13 (0.11−0.16)	9.57 (4.61−16.66)	428.42 (275.30−627.83)	0.11 (0.09−0.13)
**Northwest**						
Gansu	22.37 (10.79−40.09)	916.15 (573.96−1380.27)	0.14 (0.12−0.17)	9.17 (4.11−16.91)	477.58 (327.65−703.22)	0.12 (0.10−0.14)
Ningxia	17.00 (7.82−29.71)	736.80 (475.49−1109.17)	0.13 (0.11−0.16)	6.31 (2.74−11.94)	401.84 (283.29−576.25)	0.12 (0.10−0.14)
Qinghai	23.67 (11.64−39.42)	999.56 (645.51−1473.99)	0.14 (0.12−0.18)	11.66 (5.67−21.89)	595.93 (418.23−880.16)	0.13 (0.11−0.16)
Shaanxi	26.48 (11.29−48.20)	987.97 (553.18−1561.67)	0.14 (0.11−0.17)	9.70 (3.71−18.74)	475.97 (297.94−721.62)	0.12 (0.10−0.14)
Xinjiang	32.50 (16.29−56.10)	1228.56 (706.45−1956.42)	0.15 (0.12−0.18)	9.13 (3.78−17.72)	463.90 (297.93−716.19)	0.12 (0.10−0.14)
**Southwest**						
Chongqing	32.96 (14.56−59.19)	1224.37 (699.12−1870.75)	0.11 (0.09−0.12)	13.30 (5.76−25.41)	593.84 (370.51−941.62)	0.16 (0.13−0.19)
Guizhou	29.95 (16.87−48.03)	1219.81 (820.72−1724.10)	0.17 (0.13−0.21)	14.81 (7.93−24.82)	714.59 (506.57−1009.36)	0.17 (0.14−0.21)
Sichuan	34.21 (14.50−61.89)	1224.85 (641.80−1940.45)	0.26 (0.21−0.31)	14.27 (5.19−28.63)	617.24 (351.37−1025.07)	0.18 (0.15−0.21)
Xizang	32.66 (15.46−58.84)	1298.35 (735.83−2128.88)	0.12 (0.10−0.15)	16.55 (7.77−31.35)	739.02 (450.86−1219.47)	0.13 (0.10−0.15)
Yunnan	26.78 (15.96−42.68)	1221.14 (867.59−1682.87)	0.24 (0.20−0.28)	14.08 (8.07−23.02)	785.51 (561.23−1087.72)	0.25 (0.21−0.30)
**SARs**						
Hong Kong	15.98 (8.59−25.96)	643.48 (420.43−958.99)	0.13 (0.11−0.16)	6.86 (4.14−11.13)	290.53 (206.66−398.76)	0.09 (0.07−0.10)
Macao	17.87 (9.07−29.76)	781.71 (515.80−1138.74)	0.17 (0.14−0.20)	7.52 (3.84−13.67)	397.53 (270.99−560.65)	0.13 (0.11−0.15)

ASMR. Age-standardized mortality rate; DALY. Disability-adjusted life year; GDP. Gross domestic product; SARs. Special administrative regions; UI. Uncertainty interval

When comparing the two key time points, the number of deaths attributable to high alcohol use was 245.89 thousand (95% UI 120.55−425.82) in 1990 and 233.98 thousand (95% UI 97.49−430.07) in 2023, and the number of DALYs was 10,482.76 thousand (95% UI 6091.92−15,623.64) in 1990 and 9594.00 thousand (95% UI 5600.20−15,108.25) in 2023, and provincial values are listed in **Additional file 1:** Table S3. From the perspective of indirect economic burden, the absolute cost was USD 607.87 million (95% UI 495.54−722.08) in 1990 and USD 24,707.05 million (95% UI 20,236.93−28,588.36) in 2023, and provincial values are listed in **Additional file 1:** Table S3. Based on 1000 paired Monte Carlo draws for indirect economic burden in 1990 and 2023, the paired difference was statistically significant (paired *t*-test: *P*<0.001; Wilcoxon signed-rank test: *P*<0.001). Additionally, one-way uncertainty analyses of all parameters for 1990 and 2023 indicated that the uncertainty of YLDs and deaths had the greatest influence on the results (0.09%−0.23% in 1990; 0.08%−0.21% in 2023), followed by the proportion of labor income in GDP (0.13%−0.18% in 1990; 0.11%−0.16% in 2023) (**Additional file 1:** Table S4).

From 1990 to 2023, the trends and corresponding EAPCs for five key indicators of all-cause burden attributable to high alcohol use are presented in Table 2. The national ASMR (EAPC=−3.02%, 95% UI −4.15 to −1.85, *P*<0.01), age-standardized DALY rate (EAPC=−2.26%, 95% UI −3.05 to −1.52, *P*<0.01), and the overall indirect economic burden as a percentage of GDP (EAPC=−0.47%, 95% UI −0.77 to −0.17, *P*<0.01) all demonstrated a significant decreasing trend. Regarding the 2 sub-indicators of indirect economic burden, productivity loss due to deaths as a percentage of GDP showed a significant decreasing trend (EAPC=−1.69%, 95% UI −2.25 to −1.14, *P*<0.01). At the provincial level, ASMR and age-standardized DALY rates decreased significantly (EAPC<0, *P<*0.01) (Table 2). The overall indirect economic burden as a percentage of GDP decreased in most provinces, with no statistically significant increases observed (Table 2). The composition patterns of indirect economic burden varied across provinces. For productivity loss due to deaths, the percentage of GDP showed declining EAPCs in all provinces (EAPC<0) (Table 2). However, productivity loss due to morbidity as a percentage of GDP showed a significant increasing trend (*P<*0.05) in more than one-third of the provinces.

**Table 2:** EAPC of high alcohol use attributable all causes ASMR, DALY rate, and productivity loss of deaths as a percentage of GDP, productivity loss of morbidity as a percentage of GDP, indirect economic burden as a percentage of GDP in China, 1990−2023.

**Parameter**	**EAPC of Age-standardized rate (95% UI)**				**EAPC of GDP (%, 95% UI)**					
	**Death**	***P*****-value**	**DALY**	***P*****-value**	**Productivity loss due to deaths**	***P*****-value**	**Productivity loss due to morbidity**	***P*****-value**	**Indirect economic burden**	***P*****-value**
**China**	−3.02 (−4.15 to −1.85)	<0.01	−2.26 (−3.05 to −1.52)	<0.01	−1.69 (−2.25 to −1.14)	<0.01	0.18 (−0.06 to 0.43)	0.144	−0.47 (−0.77 to −0.17)	<0.01
**Northeast**										
Heilongjiang	−3.26 (−4.54 to −1.93)	<0.01	−2.40 (−3.34 to −1.47)	<0.01	−0.99 (−1.58 to −0.44)	<0.01	0.03 (−0.22 to 0.27)	0.808	−0.31 (−0.62 to −0.01)	<0.05
Jilin	−3.65 (−4.77 to −2.61)	<0.01	−2.65 (−3.46 to −1.84)	<0.01	−1.39 (−1.98 to −0.83)	<0.01	−0.36 (−0.63 to −0.11)	<0.01	−0.72 (−1.03 to −0.41)	<0.01
Liaoning	−2.43 (−3.65to −1.14)	<0.01	−1.78 (−2.66 to −0.85)	<0.01	−1.23 (−1.80 to −0.64)	<0.01	0.02 (−0.25 to 0.26)	0.884	−0.45 (−0.76 to −0.14)	<0.01
**North**										
Beijing	−3.71 (−4.89 to −2.52)	<0.01	−2.07 (−2.81 to −1.32)	<0.01	−2.06 (−2.69 to −1.47)	<0.01	0.15 (−0.08 to 0.43)	0.276	−0.26 (−0.53 to 0.01)	0.054
Hebei	−2.22 (−3.38 to −0.92)	<0.01	−1.82 (−2.70 to −0.92)	<0.01	−1.49 (−2.01 to −0.90)	<0.01	0.26 (0.01 to 0.52)	<0.05	−0.38 (−0.67 to −0.08)	<0.05
Inner Mongolia	−3.38 (−4.77 to −1.95)	<0.01	−2.22 (−3.03 to −1.37)	<0.01	−0.97 (−1.54 to -0.43)	<0.05	−0.01 (−0.25 to 0.25)	0.936	−0.24 (−0.50 to 0.02)	0.080
Shanxi	−3.38 (−4.50 to −2.16)	<0.01	−2.42 (−3.20 to −1.65)	<0.01	−1.96 (−2.57 to −1.39)	<0.01	0.10 (−0.17 to 0.35)	0.468	−0.53 (−0.83 to −0.23)	<0.01
Tianjin	−3.00 (−4.38 to −1.56)	<0.01	−1.97 (−2.81 to −1.06)	<0.01	−1.82 (−2.46 to −1.23)	<0.01	−0.26 (−0.52 to 0.01)	0.062	−0.63 (−0.93 to −0.33)	<0.01
**East**										
Anhui	−3.62 (−4.81 to −2.37)	<0.01	−2.83 (−3.69 to −2.01)	<0.01	−1.80 (−2.37 to −1.21)	<0.01	−0.08 (−0.36 to 0.18)	0.506	−0.69 (−0.97 to −0.38)	<0.01
Fujian	−3.44 (−4.68 to −2.19)	<0.01	−2.52 (−3.31 to −1.80)	<0.01	−1.84 (−2.46 to −1.21)	<0.01	0.68 (0.43 to 0.93)	<0.01	−0.20 (−0.52 to 0.11)	0.206
Jiangsu	−2.58 (−3.82 to −1.30)	<0.01	−2.22 (−3.02 to −1.42)	<0.01	−1.92 (−2.48 to −1.33)	<0.01	−0.30 (−0.54 to −0.04)	<0.05	−0.86 (−1.17 to −0.54)	<0.01
Jiangxi	−3.00 (−4.04 to −1.92)	<0.01	−2.40 (−3.14 to −1.63)	<0.01	−1.63 (−2.19 to −1.07)	<0.01	0.28 (0.04 to 0.52)	<0.05	−0.45 (−0.74 to -0.12)	<0.01
Shandong	−3.00 (−4.32 to −1.65)	<0.01	−2.16 (−2.93 to −1.33)	<0.01	−1.82 (−2.39 to −1.27)	<0.01	0.25 (0.01 to 0.49)	<0.05	−0.50 (−0.82 to −0.19)	<0.01
Shanghai	−3.68 (−4.87 to −2.43)	<0.01	−2.12 (−2.79 to −1.45)	<0.01	−2.95 (−3.53 to −2.38)	<0.01	−0.23 (−0.46 to 0.02)	0.068	−0.68 (−0.93 to −0.42)	<0.01
Zhejiang	−3.19 (−4.34 to −2.12)	<0.01	−2.55 (−3.26 to −1.81)	<0.01	−2.21 (−2.79 to −1.63)	<0.01	0.08 (−0.17 to 0.35)	0.514	−0.65 (−0.94 to −0.36)	<0.01
**South**										
Guangdong	−3.98 (−5.16 to −2.75)	<0.01	−2.79 (−3.55 to −1.99)	<0.01	−2.67 (−3.22 to −2.06)	<0.01	0.56 (0.31 to 0.80)	<0.01	−0.33 (−0.61 to -0.04)	0.020
Guangxi	−2.61 (−3.65 to −1.60)	<0.01	−2.10 (−2.83 to −1.33)	<0.01	−1.20 (−1.80 to −0.65)	<0.01	0.61 (0.35 to 0.86)	<0.01	−0.22 (−0.57 to 0.14)	0.214
Hainan	−2.79 (−3.83 to −1.82)	<0.01	−2.00 (−2.67 to −1.35)	<0.01	−0.96 (−1.60 to -0.30)	<0.01	1.01 (0.76 to 1.25)	<0.01	0.24 (-0.11 to 0.58)	0.158
**Central**										
Henan	−3.19 (−4.52 to −1.88)	<0.01	−2.25 (−3.12 to −1.35)	<0.01	−1.78 (−2.40 to −1.15)	<0.01	0.53 (0.27 to 0.79)	<0.01	−0.29 (−0.60 to 0.04)	0.088
Hubei	−3.55 (−4.76 to −2.30)	<0.01	−2.71 (−3.57 to −1.83)	<0.01	−1.56 (−2.12 to −1.01)	<0.01	−0.01 (−0.22 to 0.23)	0.912	−0.52 (−0.80 to −0.23)	<0.01
Hunan	−3.37 (−4.31 to −2.32)	<0.01	−2.57 (−3.27 to −1.84)	<0.01	−1.81 (−2.38 to −1.22)	<0.01	−0.04 (−0.29 to 0.19)	0.722	−0.77 (−1.11 to −0.43)	<0.01
**Northwest**										
Gansu	−2.90 (−4.06 to −1.80)	<0.01	−2.13 (−2.82 to −1.44)	<0.01	−1.51 (−2.15 to −0.85)	<0.01	−0.30 (−0.56 to −0.05)	<0.05	−0.67 (−1.01 to −0.36)	<0.01
Ningxia	−3.40 (−4.60 to −2.22)	<0.01	−2.15 (−2.77 to −1.49)	<0.01	−1.90 (−2.53 to −1.27)	<0.01	0.41 (0.16 to 0.64)	<0.01	−0.33 (−0.63 to -0.01)	0.038
Qinghai	−2.15 (−3.20 to −1.07)	<0.01	−1.59 (−2.26 to −0.94)	<0.01	−0.81 (−1.43 to -0.14)	< 0.05	0.26 (-0.01 to 0.52)	0.054	−0.11 (−0.43 to 0.22)	0.504
Shaanxi	−3.33 (−4.63 to −2.11)	<0.01	−2.49 (−3.26 to −1.71)	<0.01	−1.90 (−2.49 to −1.30)	<0.01	0.12 (−0.13 to 0.37)	0.304	−0.56 (−0.88 to −0.25)	<0.01
Xinjiang	−4.01 (−5.08 to −2.83)	<0.01	−3.10 (−3.87 to −2.32)	<0.01	−2.35 (−2.94 to −1.72)	<0.01	0.16 (−0.08 to 0.41)	0.218	−0.68 (−0.98 to −0.36)	<0.01
**Southwest**										
Chongqing	−2.62 (−3.77 to −1.39)	<0.01	−2.12 (−2.98 to −1.29)	<0.01	−0.23 (−0.78 to 0.34)	0.398	0.30 (0.05 to 0.55)	<0.01	0.11 (−0.22 to 0.42)	0.478
Guizhou	−1.84 (−2.67 to −0.92)	<0.01	−1.32 (−1.96 to −0.71)	<0.01	−0.61 (−1.22 to -0.01)	< 0.05	1.04 (0.79 to 1.29)	<0.01	0.31 (−0.06 to 0.66)	0.120
Sichuan	−2.60 (−3.95 to −1.29)	<0.01	−2.12 (−3.02 to −1.19)	<0.01	−1.81 (−2.42 to −1.25)	<0.01	−0.54 (−0.77 to −0.31)	<0.01	−1.08 (−1.39 to −0.78)	<0.01
Xizang	−2.06 (−3.10 to −0.91)	<0.01	−1.80 (−2.55 to −1.01)	<0.01	−0.71 (−1.29 to −0.11)	<0.05	0.43 (0.14 to 0.71)	<0.01	−0.05 (−0.41 to 0.31)	0.736
Yunnan	−1.87 (−2.73 to −1.00)	<0.01	−1.25 (−1.80 to −0.71)	<0.01	−0.55 (−1.16 to 0.05)	0.084	0.51 (0.26 to 0.75)	<0.01	0.15 (−0.14 to 0.46)	0.376
**SARs**										
Hong Kong	−2.02 (−2.92 to −1.14)	<0.01	−2.08 (−2.70 to −1.45)	<0.01	−1.69 (−2.28 to −1.13)	<0.01	−1.01 (−1.25 to −0.77)	<0.01	−1.26 (−1.54 to −0.94)	<0.01
Macao	−1.99 (−2.98 to −0.91)	<0.01	−1.69 (−2.41 to −1.07)	<0.01	−1.47 (−2.09 to −0.84)	<0.01	−0.25 (−0.49 to -0.01)	<0.05	−0.63 (−0.92 to −0.32)	<0.01

DALY. Disability-adjusted life year; EAPC. Estimated annual percentage change; GDP. Gross domestic product; SARs. Special administrative regions; UI. Uncertainty interval

### Sub-national variations

**Fig. 1:**
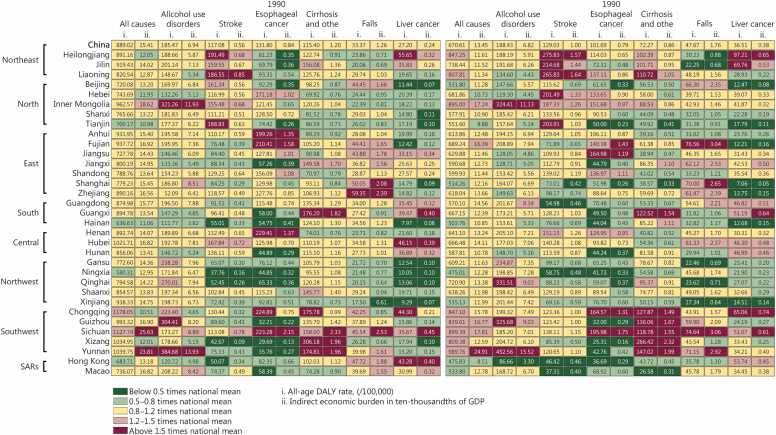
National and sub-national burden attributable to high alcohol use: All-age disability-adjusted life year (DALY) rate for all causes and six specific diseases, and indirect economic burden in ten-thousandths of GDP.

Substantial provincial heterogeneity was observed in both the all-cause DALY rate and the indirect economic burden as a percentage of GDP attributable to high alcohol use. In 1990, provinces with relatively high all-cause DALY rates were concentrated mainly in Southwest China, whereas provinces with relatively high indirect economic burden were concentrated in the Southwest and East China (Fig. 1). By 2023, the spatial pattern of disease burden remained broadly similar, with persistently high levels in Southwest China; several provinces in Northeast China, particularly Heilongjiang and Liaoning, also shifted toward relatively higher levels. The distribution of indirect economic burden was likewise largely stable over time, although some provinces, such as Chongqing, shifted from relatively low to relatively high levels (Fig. 1).

Patterns also varied across specific causes. In 2023, the DALY burden from alcohol use disorders attributable to high alcohol use remained concentrated in Guizhou, Yunnan, Inner Mongolia, and Qinghai; stroke was more prominent in the Northeast and parts of North China; esophageal cancer in Jiangsu, Chongqing, and Sichuan; cirrhosis and other chronic liver diseases in Southwest China, Guangxi, and Liaoning; falls in Fujian and Sichuan; and liver cancer in Jilin, Heilongjiang, and Chongqing. For each cause, the spatial distribution of indirect economic burden generally overlapped with, but did not fully mirror, the distribution of disease burden. Detailed province-level comparisons are provided in **Additional file 1:** Tables S5-S10.

Most provinces showed broadly concordant rankings between disease burden and indirect economic burden (**Additional file 1:** Table S9 **and** Table S10), although geographic mismatches remained. In 2023, provinces with relatively higher disease burden than indirect economic burden included Heilongjiang, Liaoning, Hebei, Tianjin, Chongqing, and Xizang, whereas provinces with relatively higher indirect economic burden included Beijing, Fujian, Shanghai, Hainan, Ningxia, Xinjiang, Yunnan, and Macao (**Additional file 1:** Table S10). Compared with 1990, these mismatches became less geographically clustered. In 1990, provinces with relatively higher disease burden were Heilongjiang, Hebei, Chongqing, and Xizang, whereas those with relatively higher indirect economic burden were Inner Mongolia, Ningxia, Sichuan, Yunnan, and Hong Kong (SAR) (**Additional file 1:** Table S9).

### Sex and age differences

**Fig. 2:**
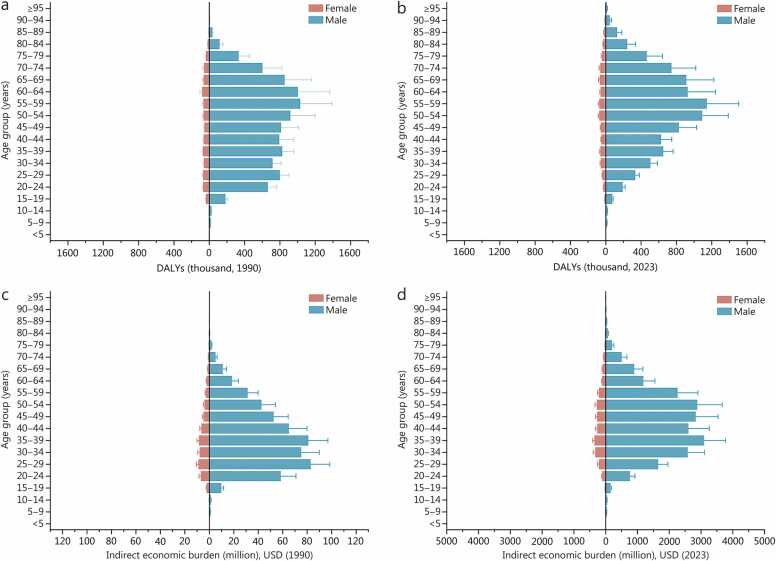
Distribution of high alcohol use attributable to all causes disability-adjusted life years (DALYs) and indirect economic burden by sex and age group in 1990 and 2023. **a** Distribution of high alcohol use attributable to all causes DALYs by sex and age group in 1990. **b** Distribution of high alcohol use attributable to all causes DALYs by sex and age group in 2023. **c** Indirect economic burden by sex and age group in 1990. **d** Indirect economic burden by sex and age group in 2023. USD. United States dollar.

As shown in Fig. 2, from a sex perspective, the burden associated with high alcohol use was highly concentrated among males. Across age groups, DALYs generally increased from younger ages to late middle age. For males, DALYs peaked at 55−59 years in both 1990 and 2023. However, for females, the peak occurred at 60−64 years in 1990 and shifted to 50−54 years in 2023. In older age groups, DALYs declined for both sexes. The indirect economic burden was concentrated in working-age adults, with the peak shifting from 25−29 years in 1990 to 35−39 years in 2023. Detailed sex- and age-specific estimates are provided in **Additional file 1:** Tables S11-S12.

### Forecasting

**Fig. 3:**
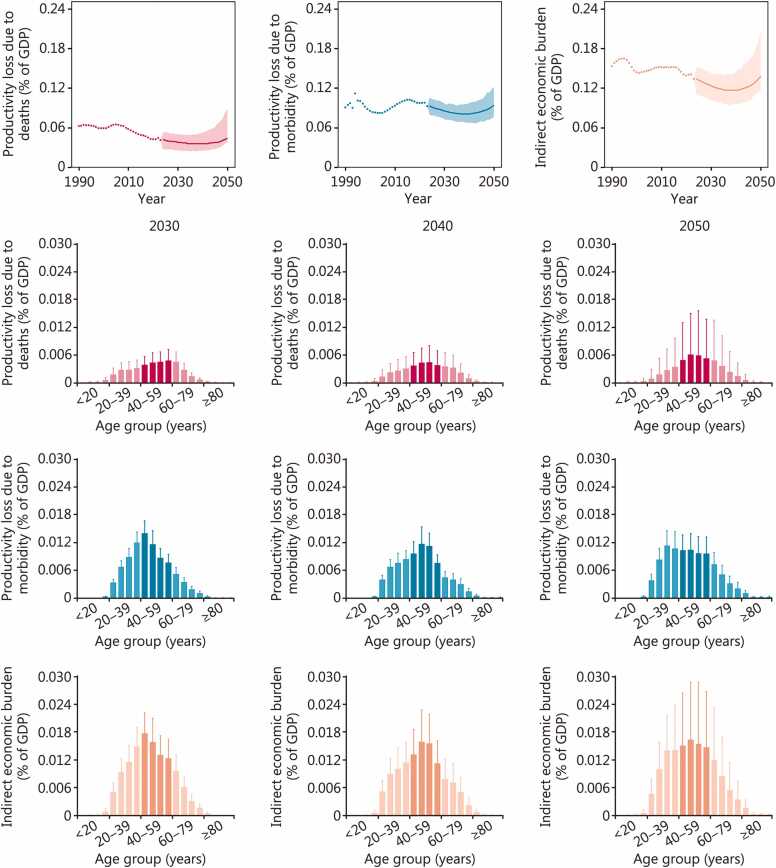
Forecasting for high alcohol use attributable to indirect economic burden by age groups from 2024 to 2050. The dotted line plot shows forecasting for the productivity loss of deaths as a percentage of gross domestic product (GDP), productivity loss of morbidity as a percentage of GDP, and indirect economic burden as a percentage of GDP from 2024 to 2050. The bar chart shows forecasting for productivity loss of deaths as a percentage of GDP, productivity loss of morbidity as a percentage of GDP, and indirect economic burden as a percentage of GDP by age groups in 2030, 2040, and 2050.

The performance of the forecasting procedure was assessed using RMSE and MAPE (**Additional file 1:** Table S13). As shown in Fig. 3 and **Additional file 1:** Table S14, forecasting indicates that from 2024 to 2050, the indirect economic burden attributable to high alcohol use in China will exhibit a trend of initial decline followed by an increase. The overall indirect burden is forecast to reach its lowest level during the forecast period in 2039 [0.12% (95% UI 0.10−0.14)] before rebounding, with the level in 2050 [0.14% (95% UI 0.12−0.21)] slightly exceeding the starting point in 2024 [0.13% (95% UI 0.11−0.15)]. In terms of composition, productivity loss due to morbidity consistently dominates the total burden throughout the forecast period (Fig. 3**; Additional file 1:** Table S14).

Age-specific forecasts of disease burden (deaths and YLDs) and indirect economic burden are presented in **Additional file 1:** Tables S15-S16. Across age groups, the indirect economic burden is concentrated in working-age adults, while the contribution from older age groups shows an overall upward tendency over the projection horizon, consistent with population ageing. In contrast, younger age groups contribute minimally and show a decreasing tendency by the end of the forecast period (**Additional file 1:** Tables S15-S16).

## Discussion

This study systematically evaluated the disease burden and indirect economic burden attributable to high alcohol use in China from 1990 to 2023. Nationally, relative metrics, including ASMR, age-standardized DALY rate, and the indirect economic burden, demonstrated a synchronized and significant decline over the past three decades. However, a striking disparity emerged in absolute terms: while the absolute numbers of deaths and DALYs decreased only marginally, the absolute indirect economic loss surged exponentially. Furthermore, beneath the overall declining trend, productivity loss attributable to morbidity is conversely rising. This divergence highlights that while public health interventions may have mitigated fatal outcomes, the chronic non-fatal impacts of alcohol use continue to exert mounting pressure on the labor force.

The pronounced spatial heterogeneity observed in this study, particularly the divergence between regions with predominantly high disease burden (e.g., the Northeast and North China) and those with high indirect economic burden (e.g., the East China), suggests that precision prevention is a promising direction for optimizing future interventions [34]. In this context, it may be beneficial for policy frameworks to transition towards strategies tailored to specific provincial profiles. For instance, in regions with high disease burdens, efforts could prioritize healthcare resource allocation, early screening, and alcohol use disorders treatment to mitigate physical harm [35]; conversely, in regions with high economic burdens, emphasizing workplace-based interventions and productivity protection might be more effective in reducing indirect financial losses [36]. Adapting measures to these distinct regional characteristics could offer a valuable reference for maximizing the cost-effectiveness of alcohol control strategies in China.

Regarding the six leading Level-3 alcohol-attributable causes examined, we observed marked provincial heterogeneity and, in several provinces, discordance between relative disease burden and relative indirect economic burden. In 2023, the Northeast and North China more often exhibited relatively higher disease burden, whereas relatively higher indirect economic burden was also observed in parts of East China, while the Southwest consistently remained at elevated levels in both dimensions. These patterns may be influenced by differences in economic output, labor-force participation, and population age structure, and suggest the potential value of province-tailored alcohol-control strategies that jointly consider health loss and productivity loss. In addition, the burden was highly concentrated among men, and the peak of economic loss shifted toward middle-aged groups over time. These patterns align with sex differences in alcohol exposure and imply that gender-responsive strategies (e.g., targeted messaging, improved treatment access, and workplace programs in male-dominated industries) are critical [37], [38].

Compared with previous national and international studies [6], [8], the indirect economic burden estimated in this study was lower, as we focused on productivity loss, whereas earlier studies incorporated both direct and indirect costs. A previous Shanghai-based study in 2020 reported estimates of alcohol-attributable indirect burden largely consistent with the regional results for Shanghai in this study [8]. Differences across studies primarily arise from methodological approaches and inconsistencies in cost definitions.

Previous studies suggest that the disease burden attributable to high alcohol use will decline [39], [40]. However, forecasting for the indirect economic burden remains limited. The forecasts in this study indicate that the indirect economic burden will first decrease and then increase, with adults aged 40−59 years contributing most of the indirect burden during this period. Alcohol-related productivity losses will continue to affect China’s social and economic development. Targeted interventions for middle-aged adults are therefore essential to mitigate these losses [40].

This study has several limitations related to the forecasting methodology and the data used. Regarding the forecasting methodology, although the BAPC model is a robust and widely accepted tool for projecting disease burden based on demographic and historical trends, it differs from the ensemble modeling approach used by the Institute for Health Metrics and Evaluation. The ensemble approach incorporates a wide array of covariates (e.g., sociodemographic changes, risk exposure levels, and policy interventions); however, reliable long-term forecasts for these specific covariates are currently unavailable. Consequently, the forecasting in this study assumes that historical trends in the effects of age, period, and cohort will continue, and does not explicitly model the potential impact of unforeseen future policy shifts or rapid technological advancements in healthcare. In addition, the forecasts of the indirect economic burden rely on externally projected macroeconomic and demographic inputs (including future GDP and age-specific probabilities of death) sourced from GBD resources and related studies. To mitigate this, we conducted extensive sensitivity analyses, including wide-ranging one-way analyses and 1000 Monte Carlo simulations that incorporate nearly all model parameters, and we report 95% UIs from these simulations. For some inputs, publicly available releases may provide limited uncertainty information (such as probabilities of death with only point estimates) and may lead to underestimation of forecast uncertainty. Residual uncertainty may remain due to unquantified uncertainty in the exogenous projections and unforeseeable future shocks.

## Conclusions

Although the age-standardized health burden attributable to high alcohol use in China has declined from 1990 to 2023, alcohol use continues to generate substantial health and productivity losses. The burden is not evenly distributed, with clear disparities across provinces and a disproportionate impact on men and working-age adults. Moreover, the persistence of indirect economic loss in future projections suggests that alcohol-related harm will remain an important public health and economic challenge in China. For policymakers, alcohol-control policies should be tailored to provincial profiles, considering both disease burden and productivity loss. Interventions should focus on high-risk populations, particularly men and middle-aged workers, through strengthened prevention, early identification, treatment, and workplace-based programs. Continued surveillance and targeted policy action will be essential to reduce the long-term health and economic consequences of high alcohol use.

## Abbreviations

ASMR: Age-standardized mortality rate

BAPC: Bayesian age-period-cohort

COI: Cost-of-illness

DALY: Disability-adjusted life year

EAPC: Estimated annual percentage change

GBD: Global burden of disease

GDP: Gross domestic product

MAPE: Mean absolute percentage error

MR-BRT: Meta-regression—Bayesian, regularized, trimmed

PAF: Population attributable fraction

RMSE: Root-mean-square error

RR: Relative risk

SAR: Special administrative region

ST-GPR: Spatiotemporal Gaussian process regression

TMREL: Theoretical minimum risk exposure level

UI: Uncertainty interval

USD: United States dollar

YLD: Years lived with disability

## Funding

This work was supported by the Non-communicable Chronic Diseases-National Science and Technology Major Project (2024ZD0524100, 2024ZD0524101)

Availability of data and materials

The epidemiological datasets analysed during the current study are available via the Global Health Data Exchange query tool (https://vizhub.healthdata.org/gbd-results), with national datasets publicly available, provincial datasets accessible by contacting the corresponding authors, and key economic data inputs are listed in **Additional file 1:** Table S2.

## Footnotes

Appendix A Supplementary data associated with this article can be found in the online version at doi:10.1016/j.mmr.2026.100043.
