# Supplementary material for: The national and provincial disease and indirect economic burden of high alcohol use in China from 1990 to 2023 with forecasting to 2050
Source: Mil Med Res. 2026 Jun 5;13(1):100043. doi: 10.1016/j.mmr.2026.100043 (PMC13264366; doi:10.1016/j.mmr.2026.100043)
Supplement: Supplementary file 1 — Supplementary material [file mmc1.pdf]

Table S1 GBD data used to extract alcohol-attributable outcomes from the GBD Results Tool

GBD data	Details
Estimate	Measure: deaths, DALYs, YLDs<br>Metric: number, rate
Risk factor	High alcohol use
Probability of death	Measure: PoD (probability of death)<br>Metric: probability of death
Cause	All causes;<br>Alcohol use disorders;<br>Stroke;<br>Esophageal cancer;<br>Cirrhosis and other chronic liver diseases;<br>Falls;<br>Liver cancer
Population	Measure: Pop (Population)<br>Metric: number
Location	China
Age	All ages, age-standardized<br>Age groups: <5, 5−9, 10−14, 15−19, 20−24, 25−29, 30−34, 35−39, 40−44, 45−49, 50−54, 55−59, 60−64, 65−69, 70−74, 75−79, 80−84, 85−89, 90−94, >95
Sex	Both, male, female
Year	1990−2023

This table summarizes the extraction settings used in the GBD Results Tool to obtain high alcohol use-attributable epidemiological outcomes (deaths, DALYs, and YLDs) and model inputs (probability of death and population) for China from 1990 to 2023, stratified by cause, year, age group, and sex. Extracted outcomes were used for descriptive burden analyses, while deaths, YLDs, probability of death, and population were additionally used as inputs for estimating the indirect economic burden. GBD. Global Burden of Disease; DALYs. Disability-adjusted life years; YLDs. Years lived with disability

Table S2 Summary of data sources, key parameters, and the rationales for estimating indirect economic burden

Category	Variable	Mean	Range (Distribution)	Rationale	Source	Reference
Epidemiological inputs	High alcohol use-related Deaths	-	(Lower-upper) (Lognormal)	Productivity loss of deaths caused by high alcohol use	GBD 2023; Briggs *et al.* (2006)	[1,2]
	High alcohol use-related YLDs	-	(Lower-upper) (Lognormal)	Productivity loss of morbidity caused by high alcohol use		
	Probability of death (Both, Male, Female) (1990−2023)	-	-	Age-specific probability of death is needed to estimate the counterfactual survival probabilities to each age if premature deaths had not occurred		
Economic data inputs	Regional per capita GDP (1990−2023)	-	-	Per capita GDP of China and its provinces, 1990−2023: used to calculate indirect economic burden	China Statistical Yearbook 2024	[3]
	Total GDP (1990−2023)	-	-	GDP of China and its provinces, 1990−2023: used to calculate the indirect economic as a percentage of GDP		
	Age-specific labor force participation rates	-	±10% (Beta)	Labor force participation rates are needed to estimate productivity loss of morbidity caused by high alcohol use	The Seventh National Population Census of China	[4]
	The share of labor income in GDP	59.22%	50.00%−70.00% (Beta)	Per capita labor income is calculated based on per capita GDP	The Penn World Table database; Bai *et al.* (2010)	[5,6]
Model parameters and assumptions	The value of household productivity	30.00%	±50% (Triangular)	The literature notes the existence of household production among the unemployed, but also indicates that a large number of unemployed individuals are unlikely to simultaneously engage in household production	Burda *et al.* (2010)	[7]
	The income conversion ratio for household production	50.00%	±50% (Triangular)	The literature indicates that the income level from household production may be lower than that of individuals participating in the formal labor force	Biddle *et al.* (2020); Burda *et al.* (2010)	[7,8]
	Annual wage growth	5.00%	3.00%−8.00%	Considering recent wage growth, using an approximate	China Statistical	[3]

Category	Variable	Mean	Range (Distribution)	Rationale	Source	Reference
	rate		(Triangular)	5% annual wage growth rate is a reasonable assumption	Yearbook 2024	
	Discount rate	5.00%	3.00%−8.00% (Triangular)	A 5% discount rate is widely adopted in health economic research in China	Fang H *et al.* (2022); Drummond *et al.* (2003)	[9,10]
	Simulated terminal age	100.00	-	Near-lifetime simulation horizons of this duration (100 years) have been widely applied in assessing the indirect economic burden	Zhou D *et al.* (2025)	[11]
Forecasting (2024−2050)	Forecasted per capita GDP	-	(Lower-upper) (Lognormal)	For forecasting the indirect economic burden from 2024 to 2050	Global Burden of Disease Collaborative Network (2024)	[12]
	Forecasted probability of death	-	-	For forecasting survival probabilities (2024−2050)	Global Burden of Disease Collaborative Network (2024)	[13]

This table lists the epidemiological and economic inputs, model parameters, and forecasting inputs used in the cost-of-illness framework, along with their sources, assumed distributions, and uncertainty ranges. Parameter ranges were used for one-way sensitivity analyses, and specified distributions were jointly sampled in Monte Carlo simulations to quantify uncertainty in indirect economic burden estimates. GBD. Global Burden of Disease; GDP. Gross domestic product; YLDs. Years lived with disability

Table S3 Deaths, DALYs, and indirect economic burden (million) attributable to high alcohol use in China in 1990 and 2023

Parameter	1990 (95% UI)			2023 (95% UI)		
	Disease burden (thousand)		Indirect economic burden (million)	Disease burden (thousand)		Indirect economic burden (million)
	Deaths	DALYs		Deaths	DALYs	
**China**	245.89 (120.55−425.82)	10,482.76 (6091.92−15,623.64)	607.87 (495.54−722.08)	233.98 (97.49−430.07)	9594.00 (5600.20−15,108.25)	24,707.05 (20,236.93−28,588.36)
**Northeast**						
Heilongjiang	7.17 (2.83−13.55)	323.05 (177.92−519.07)	18.02 (14.64−21.28)	6.58 (2.27−13.26)	254.40 (129.64−437.68)	271.28 (213.59−305.67)
Jilin	5.37 (2.62−9.43)	232.60 (138.27−358.98)	12.47 (10.29−14.90)	4.27 (1.89−7.97)	170.53 (103.80−272.70)	227.85 (183.41−262.28)
Liaoning	8.06 (3.31−14.68)	330.98 (182.11−515.54)	28.58 (22.77−33.65)	9.57 (3.62−18.31)	338.25 (187.55−562.90)	505.26 (397.79−580.42)
**North**						
Beijing	1.85 (0.75−3.41)	79.56 (46.11−118.37)	13.82 (11.49−15.87)	2.39 (1.00−4.24)	115.55 (77.30−169.88)	757.83 (630.60−862.73)
Hebei	11.88 (4.69−20.96)	460.49 (245.67−712.64)	22.40 (18.13−26.36)	15.35 (5.85−28.79)	510.67 (256.29−837.95)	695.47 (557.60−779.16)
Inner Mongolia	4.12 (1.46−7.48)	212.66 (126.20−315.08)	12.43 (10.35−14.25)	4.45 (1.31−8.78)	211.61 (122.80−343.04)	612.23 (496.71−690.71)
Shanxi	5.12 (2.31−8.77)	226.94 (136.01−337.52)	11.86 (9.67−14.04)	4.73 (1.92−9.06)	199.41 (122.06−305.08)	402.83 (329.20−475.20)
Tianjin	1.53 (0.52−3.00)	63.07 (34.89−99.62)	7.14 (5.82−8.17)	1.89 (0.68−3.50)	75.99 (47.27−117.91)	216.93 (172.62−246.92)

Parameter	1990 (95% UI)			2023 (95% UI)		
	Disease burden (thousand)		Indirect economic burden (million)	Disease burden (thousand)		Indirect economic burden (million)
	Deaths	DALYs		Deaths	DALYs	
**East**						
Anhui	12.57 (5.56−21.80)	534.02 (301.26−807.25)	21.19 (16.01−22.67)	9.68 (3.84−18.56)	378.44 (220.37−596.18)	854.04 (709.95 − 999.36)
Fujian	6.48 (2.85−11.37)	290.01 (165.54−439.52)	18.48 (14.90−21.77)	6.21 (2.41−11.17)	294.17 (178.82−438.97)	1274.31 (1063.08−1486.50)
Jiangsu	11.58 (5.09−21.06)	501.30 (295.27−751.30)	42.73 (35.23−50.30)	15.59 (6.26−29.51)	540.97 (303.63−861.58)	2129.38 (1719.06−2447.01)
Jiangxi	7.26 (3.80−11.92)	309.40 (196.42−448.74)	13.40 (10.96−16.14)	6.59 (2.95−11.76)	267.72 (157.73−411.32)	590.23 (488.69−693.14)
Shandong	16.83 (6.86−29.48)	675.94 (369.07−1030.92)	43.10 (34.66−50.45)	15.98 (5.13−30.51)	607.50 (327.89−970.34)	1529.89 (1216.10−1765.42)
Shanghai	2.58 (1.03−4.66)	107.32 (62.35−160.00)	25.25 (21.57−29.37)	2.58 (1.21−4.42)	126.54 (89.68−174.91)	886.76 (754.21−1018.86)
Zhejiang	9.59 (4.49−16.73)	384.38 (224.69−571.29)	31.32 (26.13−37.05)	10.48 (4.92−18.59)	413.61 (267.79−609.32)	1659.32 (1397.88−1933.93)
**South**						
Guangdong	12.40 (5.53−21.87)	561.95 (331.03−840.87)	51.41 (42.49−60.51)	13.13 (5.50−24.37)	744.86 (479.40−1114.77)	2850.12 (2413.11−3310.84)
Guangxi	9.41 (5.03−15.21)	385.66 (243.05−564.79)	12.71 (10.20−16.13)	8.48 (3.81−15.15)	339.55 (202.78−526.02)	483.70 (395.20−581.05)
Hainan	0.97 (0.51−1.52)	43.39 (28.07−62.55)	2.37 (1.93−2.98)	1.09 (0.52−1.90)	52.63 (34.44−77.40)	116.87 (96.76−140.64)

Parameter	1990 (95% UI)			2023 (95% UI)		
	Disease burden (thousand)		Indirect economic burden (million)	Disease burden (thousand)		Indirect economic burden (million)
	Deaths	DALYs		Deaths	DALYs	
**Central**						
Henan	19.26 (7.78−36.51)	775.81 (401.28−1257.98)	27.49 (24.02−35.63)	15.93 (5.18−31.90)	646.11 (361.75−1019.51)	1138.85 (942.84 − 1310.25)
Hubei	14.41 (7.08−25.61)	563.40 (331.61−859.08)	28.98 (24.01−34.62)	9.38 (2.88−18.07)	386.06 (210.59−602.17)	1137.32 (943.31−1,306.72)
Hunan	13.26 (7.08−21.73)	531.49 (345.35−761.28)	20.87 (17.42−25.68)	10.17 (4.63−18.18)	389.13 (236.31−600.68)	775.33 (645.14−930.69)
**Northwest**						
Gansu	3.34 (1.66−5.79)	179.11 (119.29−253.91)	7.29 (5.87−8.64)	3.29 (1.34−6.24)	150.67 (96.51−234.12)	203.79 (170.13−244.16)
Ningxia	0.48 (0.25−0.81)	28.12 (19.38−39.02)	1.76 (1.44−2.12)	0.56 (0.23−1.06)	35.26 (23.88−50.52)	93.52 (78.41−109.85)
Qinghai	0.67 (0.37−1.05)	37.03 (26.15−51.45)	2.08 (1.73−2.58)	0.85 (0.40−1.55)	43.08 (29.18−63.94)	73.09 (61.26−87.94)
Shaanxi	6.36 (2.88−11.18)	287.13 (169.06−431.57)	11.69 (9.60−14.25)	5.90 (2.14−11.73)	253.28 (147.14−403.45)	577.52 (478.30−679.28)
Xinjiang	3.17 (1.61−5.44)	146.27 (87.47−220.79)	8.06 (6.75−9.79)	2.77 (1.07−5.41)	145.74 (91.36−227.55)	333.56 (277.65−389.98)
**Southwest**						
Chongqing	4.30 (1.98−7.50)	180.83 (107.03−270.03)	7.20 (5.90−8.53)	7.52 (3.14−14.49)	284.05 (164.07−465.53)	685.38 (565.14−814.19)

Parameter	1990 (95% UI)			2023 (95% UI)		
	Disease burden (thousand)		Indirect economic burden (million)	Disease burden (thousand)		Indirect economic burden (million)
	Deaths	DALYs		Deaths	DALYs	
Guizhou	6.88 (3.97−10.70)	330.46 (232.50−450.08)	9.19 (7.33−11.39)	7.42 (3.89−12.54)	333.05 (224.72−482.68)	511.98 (428.36−629.05)
Sichuan	30.10 (13.45−53.65)	1237.89 (692.66−1898.26)	47.74 (38.53−57.28)	20.79 (7.27−43.11)	759.18 (393.48−1,330.05)	1,550.31 (1266.52−1809.05)
Xizang	0.53 (0.25−0.95)	23.55 (13.83−37.68)	0.69 (0.55−0.85)	0.64 (0.30−1.19)	31.39 (19.12−51.14)	45.09 (37.21−55.39)
Yunnan	7.41 (4.50−11.42)	396.63 (290.68−532.21)	22.48 (18.87−26.81)	8.99 (5.07−14.69)	466.13 (325.95−651.44)	1081.53 (915.51−1,283.49)
**SARs**						
Hong Kong	0.93 (0.49−1.51)	39.67 (26.16−58.83)	101.42 (82.68−120.24)	1.12 (0.67−1.86)	35.93 (24.83−52.93)	324.44 (268.62−390.05)
Macao	0.05 (0.03−0.08)	2.62 (1.87−3.70)	5.47 (4.53−6.53)	0.07 (0.04−0.14)	3.72 (2.51−5.34)	58.52 (49.08−68.20)

This table reports provincial and national high alcohol use-attributable deaths and DALYs (thousands) and the corresponding indirect economic burden (million, USD) for 1990 and 2023, with 95% uncertainty intervals (UIs). DALYs. Disability-adjusted life years; SARs. Special administrative regions; USD United States dollar

Table S4 One-way sensitivity analysis for indirect economic burden (% of GDP) attributable to high alcohol use

**Parameter**	**Indirect economic burden (% of GDP), base-case estimate (lower-upper bound)**	
	**1990**	**2023**
YLDs, deaths (lower−upper)	0.15 (0.09−0.23)	0.13 (0.08−0.21)
Labor force participation rate (±10%)	0.15 (0.14−0.17)	0.13 (0.12−0.14)
The value of household productivity (15%−45%)	0.15 (0.15−0.16)	0.13 (0.13−0.14)
The income conversion ratio for household production (25%−75%)	0.15 (0.15−0.16)	0.13 (0.13−0.14)
The proportion of labor income in GDP (50%−70%)	0.15 (0.13−0.18)	0.13 (0.11−0.16)
Annual income growth rate (3%−8%)	0.15 (0.14−0.18)	0.13 (0.13−0.15)
Discount rate (8%−3%)	0.15 (0.14−0.17)	0.13 (0.12−0.14)

Variation in each parameter influenced the indirect economic burden as a percentage of GDP. Overall, in both 1990 and 2023, the largest effect came from uncertainty in the disease burden (years lived with disability and deaths) reported by the Global Burden of Disease Study, with ranges of 0.09%−0.23% in 1990 and 0.08%−0.21% in 2023. The uncertainty ranges for all other economic parameters were narrower than these. GDP. Gross domestic product; YLD. Years lived with disability

Table S5 All-age DALY rate and indirect economic burden attributable to high alcohol use in China in 1990 (all causes and 2 diseases)

Parameters	All causes	Alcohol use disorders		Stroke	
	DALYs (/100,000, 95% UI)	DALYs (/100,000, 95% UI)	Indirect economic burden (‱ of GDP)	DALYs (/100,000, 95% UI)	Indirect economic burden (‱ of GDP)
**China**	889.02 (516.64−1325.01)	185.47 (138.43−244.40)	6.94 (5.91−7.89)	117.08 (5.24−312.63)	0.56 (0.43−0.71)
**Northeast**					
Heilongjiang	891.16 (490.82−1431.91)	188.66 (136.89−255.10)	5.87 (4.95−6.71)	191.49 (9.26−507.76)	0.68 (0.50−0.86)
Jilin	919.43 (546.55−1419.00)	201.14 (144.86−275.03)	7.13 (6.07−8.15)	159.55 (6.98−416.36)	0.67 (0.47−0.81)
Liaoning	820.54 (451.46−1278.08)	148.67 (105.18−204.64)	5.34 (4.51−6.15)	186.55 (13.86−456.31)	0.85 (0.64−1.13)
**North**					
Beijing	720.08 (417.32−1071.29)	169.97 (122.54−234.45)	6.84 (5.79−7.77)	161.34 (18.82−404.23)	0.56 (0.42−0.80)
Hebei	743.69 (396.76−1150.89)	132.26 (91.81−183.91)	5.13 (4.30−5.87)	116.99 (9.67−288.89)	0.56 (0.44−0.78)
Inner Mongolia	962.57 (571.20−1426.16)	321.26 (232.50−437.82)	11.93 (10.10−13.63)	155.48 (6.93−426.03)	0.68 (0.50−0.84)
Shanxi	765.66 (458.87−1138.75)	181.83 (132.51−243.91)	6.49 (5.46−7.45)	111.21 (6.40−290.46)	0.51 (0.38−0.66)
Tianjin	700.17 (387.26−1105.82)	177.37 (126.04−246.43)	6.22 (5.24−7.20)	188.83 (8.53−479.52)	0.63 (0.48−0.91)
**East**					

Parameters	All causes	Alcohol use disorders		Stroke	
	DALYs (/100,000, 95% UI)	DALYs (/100,000, 95% UI)	Indirect economic burden (‱ of GDP)	DALYs (/100,000, 95% UI)	Indirect economic burden (‱ of GDP)
Anhui	931.95 (525.74−1408.77)	195.58 (143.48−257.08)	7.14 (6.08−8.16)	110.17 (1.16−296.52)	0.59 (0.44−0.73)
Fujian	937.72 (535.24−1421.13)	195.95 (147.37−257.06)	7.36 (6.25−8.36)	76.48 (2.81−204.67)	0.39 (0.28−0.45)
Jiangsu	727.78 (428.66−1090.72)	146.46 (106.66−206.10)	6.09 (5.14−6.95)	84.40 (3.76−222.77)	0.45 (0.33−0.58)
Jiangxi	800.19 (507.99−1160.56)	133.36 (97.36−180.14)	5.49 (4.65−6.24)	88.34 (2.67−242.91)	0.43 (0.33−0.54)
Shandong	788.76 (430.68−1202.99)	154.23 (112.93−208.72)	5.88 (4.96−6.65)	129.25 (6.15−324.85)	0.64 (0.49−0.82)
Shanghai	779.23 (452.73−1161.72)	186.80 (132.35−260.12)	8.51 (7.20−9.72)	84.25 (4.92−196.46)	0.29 (0.21−0.43)
Zhejiang	890.16 (520.33−1322.99)	152.09 (109.92−206.75)	6.41 (5.50−7.32)	118.57 (1.66−315.79)	0.49 (0.35−0.60)
**South**					
Guangdong	874.98 (515.42−1309.27)	196.50 (141.68−267.04)	7.88 (6.73−9.06)	91.51 (0.93−240.49)	0.41 (0.31−0.54)
Guangxi	894.78 (563.90−1310.37)	147.29 (107.56−197.73)	4.85 (4.19−5.68)	96.41 (4.38−254.36)	0.48 (0.37−0.62)
Hainan	636.63 (411.94−917.88)	111.77 (82.54−151.37)	3.82 (3.18−4.33)	55.01 (2.57−135.76)	0.33 (0.28−0.51)
**Central**					
Henan	892.74 (461.75−1447.56)	189.89 (136.62−262.45)	6.68 (5.58−7.65)	132.49 (6.55−358.58)	0.65 (0.48−0.79)

Parameters	All causes	Alcohol use disorders		Stroke	
	DALYs (/100,000, 95% UI)	DALYs (/100,000, 95% UI)	Indirect economic burden (‱ of GDP)	DALYs (/100,000, 95% UI)	Indirect economic burden (‱ of GDP)
Hubei	1021.71 (601.37−1557.92)	192.78 (143.25−259.72)	7.81 (6.63−8.87)	167.84 (7.35−435.73)	0.72 (0.54−0.85)
Hunan	856.06 (556.25−1226.19)	146.72 (110.09−197.01)	5.24 (4.49−6.06)	136.11 (0.74−357.71)	0.59 (0.47−0.76)
**Northwest**					
Gansu	772.60 (514.55−1095.28)	238.28 (173.97−316.93)	7.96 (6.66−9.05)	65.07 (−0.79 to 197.69)	0.30 (0.24−0.40)
Ningxia	580.31 (400.00−805.36)	171.84 (123.68−230.05)	6.47 (5.50−7.46)	37.76 (1.16−115.40)	0.16 (0.12−0.21)
Qinghai	794.58 (560.99−1103.89)	270.01 (207.77−343.66)	7.94 (6.72−9.26)	52.45 (−2.52−151.84)	0.26 (0.20−0.35)
Shaanxi	854.57 (503.15−1284.44)	197.34 (145.32−264.02)	6.56 (5.49−7.46)	102.84 (4.40−288.59)	0.45 (0.34−0.59)
Xinjiang	938.33 (561.10−1416.37)	198.73 (145.95−257.58)	6.73 (5.75−7.72)	72.42 (−0.89 to 198.93)	0.39 (0.33−0.55)
**Southwest**					
Chongqing	1178.05 (697.25−1759.08)	223.40 (163.35−293.18)	4.65 (3.97−5.27)	130.44 (−2.03 to 347.72)	0.32 (0.29−0.48)
Guizhou	993.32 (698.88−1352.90)	304.41 (232.56−398.92)	8.20 (6.69−9.36)	89.60 (1.64−248.34)	0.41 (0.32−0.52)
Sichuan	1127.74 (631.03−1729.36)	173.27 (128.90−228.39)	8.89 (7.55−10.21)	113.08 (−0.79 to 328.19)	0.78 (0.61−0.95)
Xizang	1034.95 (607.65−1655.68)	178.66 (130.21−234.62)	5.15 (4.23−5.90)	42.67 (−7.18 to 159.68)	0.09 (0.06−0.12)

Parameters	All causes	Alcohol use disorders		Stroke	
	**DALYs (/100,000, 95% UI)**	**DALYs (/100,000, 95% UI)**	**Indirect economic burden (‱ of GDP)**	**DALYs (/100,000, 95% UI)**	**Indirect economic burden (‱ of GDP)**
Yunnan	1039.75 (762.00−1395.15)	384.68 (295.34−488.42)	13.93 (11.81−15.82)	75.33 (2.35−208.96)	0.43 (0.34−0.57)
**SARs**					
Hong Kong	683.71 (450.80−1013.74)	120.72 (81.55−176.29)	4.98 (4.17−5.65)	50.07 (2.16−123.85)	0.34 (0.26−0.44)
Macao	736.07 (524.70−1037.99)	208.22 (153.36−285.48)	8.42 (7.22−9.72)	74.37 (4.38−187.34)	0.49 (0.40−0.67)

This table reports provincial and national high alcohol use–attributable deaths and DALY rate (all age) and the corresponding indirect economic burden (‱ of GDP) for 1990, with 95% uncertainty intervals (UIs). DALYs. Disability-adjusted life years; GDP. Gross domestic product; SARs. Special administrative regions

Table S6 All-age DALY rate and indirect economic burden attributable to high alcohol use in China in 1990 (4 diseases)

Parameters	Esophageal cancer		Cirrhosis and other chronic liver diseases		Falls		Liver cancer	
	DALYs (/100,000, 95% UI)	Indirect economic burden (‱ of GDP)	DALYs (/100,000, 95% UI)	Indirect economic burden (‱ of GDP)	DALYs (/100,000, 95% UI)	Indirect economic burden (‱ of GDP)	DALYs (/100,000, 95% UI)	Indirect economic burden (‱ of GDP)
**China**	131.80 (−7.30 to 291.47)	0.84 (0.67−1.09)	115.40 (44.34−220.20)	1.20 (0.83−1.44)	33.37 (13.96−53.75)	1.26 (1.12−1.50)	27.20 (11.30−61.91)	0.24 (0.17−0.31)
**Northeast**								
Heilongjiang	81.23 (−4.18 to 178.31)	0.35 (0.28−0.46)	122.74 (46.72−222.53)	0.91 (0.64−1.11)	23.86 (10.14−40.50)	0.71 (0.65−0.87)	55.65 (20.52−128.56)	0.32 (0.22−0.41)
Jilin	69.79 (−3.43 to 148.70)	0.36 (0.29−0.47)	156.08 (58.75−294.41)	1.36 (0.93−1.64)	20.06 (8.94−35.83)	0.69 (0.63−0.85)	35.83 (15.21−85.42)	0.26 (0.20−0.36)
Liaoning	93.35 (−5.56 to 199.56)	0.54 (0.42−0.71)	125.76 (45.84−233.98)	1.24 (0.81−1.49)	29.74 (12.51−49.37)	1.03 (0.93−1.27)	19.65 (7.53−44.94)	0.16 (0.11−0.20)
**North**								
Beijing	92.29 (−5.89 to 206.56)	0.35 (0.26−0.50)	98.25 (36.78−180.50)	0.87 (0.59−1.08)	44.45 (18.39−71.41)	1.66 (1.49−2.02)	11.44 (4.71−25.48)	0.07 (0.05−0.09)
Hebei	171.18 (−9.96 to 375.73)	1.02 (0.82−1.32)	69.92 (24.45−129.23)	0.76 (0.51−0.93)	24.44 (10.23−41.15)	0.95 (0.87−1.16)	20.39 (10.23−42.14)	0.17 (0.13−0.23)
Inner Mongolia	121.45 (−6.48 to 259.65)	0.65 (0.52−0.85)	120.26 (44.71−216.16)	1.04 (0.70−1.24)	22.39 (9.39−38.39)	0.81 (0.73−1.00)	18.22 (7.05−42.28)	0.13 (0.09−0.17)
Shanxi	128.50 (−6.08 to 297.70)	0.72 (0.57−0.94)	81.52 (30.70−152.68)	0.78 (0.54−0.98)	29.03 (12.04−48.39)	1.04 (0.93−1.25)	14.80 (7.46−31.98)	0.11 (0.08−0.16)
Tianjin	74.42 (−3.83 to 169.44)	0.26 (0.19−0.36)	86.59 (32.45−160.39)	0.73 (0.48−0.91)	26.02 (11.13−43.65)	0.83 (0.73−1.00)	17.19 (7.02−39.00)	0.10 (0.06−0.13)

Parameters	Esophageal cancer		Cirrhosis and other chronic liver diseases		Falls		Liver cancer	
	**DALYs (/100,000, 95% UI)**	**Indirect economic burden (‱ of GDP)**	**DALYs (/100,000, 95% UI)**	**Indirect economic burden (‱ of GDP)**	**DALYs (/100,000, 95% UI)**	**Indirect economic burden (‱ of GDP)**	**DALYs (/100,000, 95% UI)**	**Indirect economic burden (‱ of GDP)**
**East**								
Anhui	199.26 (−9.76 to 450.17)	1.35 (1.10−1.75)	89.29 (33.23−170.26)	0.92 (0.62−1.08)	28.08 (12.03−46.68)	1.04 (0.90−1.18)	19.99 (7.03−47.98)	0.18 (0.12−0.23)
Fujian	210.41 (−12.16 to 448.54)	1.58 (1.26−2.05)	105.20 (40.49−197.26)	1.13 (0.77−1.38)	44.41 (17.14−68.86)	1.65 (1.47−1.98)	12.42 (6.02−26.54)	0.12 (0.10−0.17)
Jiangsu	127.81 (−7.46 to 281.85)	1.01 (0.78−1.35)	90.98 (34.16−166.77)	1.07 (0.72−1.32)	43.88 (18.23−69.58)	1.78 (1.60−2.13)	33.15 (12.73−77.11)	0.34 (0.23−0.45)
Jiangxi	57.26 (−2.79 to 121.57)	0.39 (0.32−0.51)	149.58 (56.04−289.75)	1.70 (1.16−2.07)	36.82 (14.98−57.58)	1.56 (1.36−1.84)	25.63 (10.39−59.71)	0.25 (0.17−0.32)
Shandong	156.09 (−8.97 to 343.31)	1.08 (0.86−1.39)	70.97 (26.10−129.36)	0.79 (0.53−0.94)	28.87 (12.11−49.20)	1.13 (1.02−1.37)	27.57 (10.45−61.15)	0.24 (0.16−0.30)
Shanghai	129.98 (−6.25 to 286.31)	0.45 (0.33−0.65)	93.11 (38.89−164.02)	0.84 (0.57−1.02)	50.05 (21.04−83.65)	2.08 (1.86−2.54)	14.79 (3.43−34.06)	0.09 (0.05−0.10)
Zhejiang	127.76 (−7.56 to 277.40)	0.85 (0.68−1.12)	106.93 (41.51−198.64)	1.12 (0.77−1.33)	59.35 (23.67−91.39)	2.39 (2.12−2.83)	14.82 (5.83−31.83)	0.12 (0.09−0.16)
**South**								
Guangdong	115.48 (−6.46 to 265.31)	0.74 (0.58−0.99)	135.34 (51.94−259.46)	1.29 (0.87−1.59)	34.00 (13.90−54.96)	1.28 (1.15−1.55)	35.45 (12.88−87.06)	0.32 (0.22−0.43)
Guangxi	58.00 (−3.98−122.19)	0.44 (0.35−0.58)	176.20 (68.64−328.80)	1.82 (1.25−2.25)	27.42 (11.76−42.78)	0.91 (0.84−1.14)	39.47 (11.42−98.09)	0.40 (0.26−0.49)
Hainan	54.75 (−2.99 to 121.02)	0.41 (0.32−0.56)	124.10 (43.89−229.35)	1.30 (0.86−1.59)	34.56 (14.23−54.94)	1.23 (1.06−1.46)	7.97 (2.47−20.95)	0.08 (0.05−0.11)

Parameters	Esophageal cancer		Cirrhosis and other chronic liver diseases		Falls		Liver cancer	
	DALYs (/100,000, 95% UI)	Indirect economic burden (‱ of GDP)	DALYs (/100,000, 95% UI)	Indirect economic burden (‱ of GDP)	DALYs (/100,000, 95% UI)	Indirect economic burden (‱ of GDP)	DALYs (/100,000, 95% UI)	Indirect economic burden (‱ of GDP)
**Central**								
Henan	229.41 (−13.74 to 522.26)	1.37 (1.12−1.77)	74.01 (27.46−137.51)	0.76 (0.52−0.92)	23.71 (10.30−39.84)	0.82 (0.72−0.95)	21.60 (9.92−44.90)	0.18 (0.13−0.23)
Hubei	125.98 (−7.41 to 296.27)	0.70 (0.57−0.89)	110.19 (39.81−205.04)	1.07 (0.75−1.28)	34.58 (14.18−53.68)	1.31 (1.14−1.55)	46.15 (16.72−102.93)	0.39 (0.27−0.48)
Hunan	44.89 (−2.46 to 98.43)	0.29 (0.23−0.37)	115.10 (44.92−218.49)	1.16 (0.80−1.41)	27.73 (11.80−46.13)	1.01 (0.90−1.22)	36.89 (13.85−85.15)	0.32 (0.23−0.41)
**Northwest**								
Gansu	76.12 (−3.69 to 177.93)	0.44 (0.35−0.58)	105.79 (38.65−194.71)	1.02 (0.69−1.25)	21.72 (9.54−37.11)	0.70 (0.63−0.86)	12.54 (5.86−29.52)	0.10 (0.07−0.13)
Ningxia	44.85 (−2.26 to 100.26)	0.32 (0.25−0.43)	95.55 (34.53−175.44)	1.03 (0.71−1.31)	21.48 (9.56−35.47)	0.70 (0.68−0.94)	10.05 (4.49−23.14)	0.10 (0.07−0.14)
Qinghai	65.33 (−3.08 to 146.63)	0.36 (0.28−0.49)	120.28 (42.90−225.70)	1.15 (0.74−1.38)	20.15 (8.98−34.45)	0.64 (0.56−0.76)	13.06 (5.42−30.71)	0.10 (0.07−0.13)
Shaanxi	115.23 (−5.38 to 271.05)	0.63 (0.50−0.82)	145.77 (53.07−280.38)	1.40 (0.95−1.75)	29.24 (12.57−46.88)	0.94 (0.83−1.14)	19.71 (8.77−45.73)	0.15 (0.11−0.21)
Xinjiang	92.81 (−4.18 to 220.00)	0.51 (0.40−0.65)	78.82 (28.72−155.56)	0.73 (0.50−0.89)	17.50 (7.67−30.27)	0.61 (0.54−0.73)	9.29 (3.69−21.93)	0.07 (0.05−0.09)
**Southwest**								
Chongqing	224.89 (−11.65 to 505.33)	0.75 (0.61−0.96)	175.78 (100.24−293.13)	0.99 (0.74−1.26)	42.25 (17.82−64.63)	0.85 (0.76−1.02)	44.30 (16.44−107.44)	0.21 (0.14−0.26)

Parameters	Esophageal cancer		Cirrhosis and other chronic liver diseases		Falls		Liver cancer	
	**DALYs (/100,000, 95% UI)**	**Indirect economic burden (‱ of GDP)**	**DALYs (/100,000, 95% UI)**	**Indirect economic burden (‱ of GDP)**	**DALYs (/100,000, 95% UI)**	**Indirect economic burden (‱ of GDP)**	**DALYs (/100,000, 95% UI)**	**Indirect economic burden (‱ of GDP)**
Guizhou	32.21 (−1.80 to 68.55)	0.22 (0.17−0.29)	135.70 (47.50−260.35)	1.42 (0.94−1.70)	37.89 (14.27−59.24)	1.24 (1.09−1.47)	15.86 (6.13−38.01)	0.14 (0.10−0.19)
Sichuan	223.28 (−11.41 to 505.20)	2.15 (1.75−2.71)	158.00 (56.14−306.11)	2.33 (1.60−2.79)	45.54 (18.94−70.29)	2.53 (2.26−2.99)	35.67 (14.47−79.41)	0.45 (0.32−0.58)
Xizang	29.69 (−1.34 to 74.01)	0.13 (0.10−0.17)	306.18 (99.93−610.87)	1.96 (1.26−2.35)	26.28 (10.05−41.60)	0.66 (0.57−0.79)	17.94 (8.89−42.10)	0.10 (0.08−0.14)
Yunnan	35.76 (−1.89 to 77.41)	0.27 (0.22−0.35)	174.81 (64.73−319.69)	1.96 (1.34−2.34)	39.98 (15.99−60.94)	1.61 (1.44−1.92)	15.20 (6.44−35.59)	0.15 (0.11−0.20)
**SARs**								
Hong Kong	82.35 (−4.50 to 166.80)	0.66 (0.51−0.89)	102.03 (36.27−177.31)	1.12 (0.71−1.29)	47.72 (19.95−77.75)	1.88 (1.64−2.21)	43.28 (15.83−98.46)	0.40 (0.25−0.49)
Macao	58.39 (−2.69 to 129.66)	0.45 (0.35−0.61)	74.28 (26.46−141.34)	0.90 (0.59−1.12)	39.69 (16.54−63.07)	1.55 (1.42−1.90)	30.99 (9.96−71.44)	0.32 (0.19−0.40)

This table reports provincial and national high alcohol use–attributable deaths and DALY rate (all age) and the corresponding indirect economic burden (‱ of GDP) for 1990, with 95% uncertainty intervals (UIs). DALYs. Disability-adjusted life years; GDP. Gross domestic product; SARs. Special administrative regions

Table S7 All-age DALY rate and indirect economic burden attributable to high alcohol use in China in 2023 (all causes and 2 diseases)

Parameters	All causes	Alcohol use disorders		Stroke	
	DALYs (/100,000, 95% UI)	DALYs (/100,000, 95% UI)	Indirect economic burden (‱ of GDP)	DALYs (/100,000, 95% UI)	Indirect economic burden (‱ of GDP)
**China**	670.61 (391.45−1056.04)	188.43 (144.17−254.99)	6.82 (5.99−8.00)	129.03 (16.67−291.81)	1.00 (0.86−1.47)
**Northeast**					
Heilongjiang	847.25 (431.76−1457.65)	188.19 (135.17−261.15)	5.91 (5.30−7.15)	275.83 (34.20−642.21)	1.57 (1.32−2.41)
Jilin	738.44 (449.51−1180.88)	191.68 (140.08−265.70)	6.26 (5.58−7.49)	214.68 (23.34−486.27)	1.44 (1.22−2.16)
Liaoning	807.81 (447.90−1344.33)	134.60 (98.81−185.11)	4.43 (3.87−5.29)	265.83 (32.73−614.19)	1.64 (1.38−2.44)
**North**					
Beijing	531.80 (355.76−781.83)	147.66 (103.39−207.94)	5.57 (4.89−6.61)	115.62 (9.68−275.17)	0.69 (0.55−1.09)
Hebei	681.86 (342.21−1118.86)	119.30 (86.44−166.30)	4.45 (3.99−5.36)	201.49 (24.29−463.83)	1.33 (1.17−1.95)
Inner Mongolia	895.00 (519.36−1450.88)	324.41 (243.25−434.52)	11.13 (9.77−13.25)	187.33 (23.80−392.98)	1.26 (1.05−1.86)
Shanxi	577.91 (353.73−884.14)	185.42 (135.32−241.17)	6.21 (5.48−7.44)	133.56 (17.18−301.13)	0.96 (0.82−1.43)
Tianjin	551.60 (343.14−855.92)	157.64 (113.84−215.02)	5.14 (4.52−6.14)	203.91 (16.97−456.97)	1.03 (0.82−1.61)
**East**					

Parameters	All causes	Alcohol use disorders		Stroke	
	DALYs (/100,000, 95% UI)	DALYs (/100,000, 95% UI)	Indirect economic burden (‱ of GDP)	DALYs (/100,000, 95% UI)	Indirect economic burden (‱ of GDP)
Anhui	613.86 (357.46−967.06)	194.15 (149.36−259.53)	6.94 (6.22−8.14)	129.64 (16.66−297.49)	1.05 (0.82−1.38)
Fujian	689.24 (418.97−1028.50)	208.89 (154.42−275.92)	7.94 (6.98−9.35)	71.89 (9.12−161.16)	0.65 (0.56−0.96)
Jiangsu	629.88 (353.53−1003.19)	128.05 (94.17−179.16)	4.86 (4.31−5.74)	109.93 (14.29−237.70)	0.84 (0.71−1.23)
Jiangxi	590.68 (348.01−907.49)	128.71 (96.64−174.94)	5.05 (4.55−6.03)	112.79 (9.67−255.89)	0.91 (0.79−1.36)
Shandong	599.93 (323.80−958.24)	153.42 (113.40−207.37)	5.56 (4.95−6.60)	139.02 (17.27−313.70)	1.19 (1.04−1.73)
Shanghai	514.26 (364.45−710.82)	164.07 (116.06−229.28)	6.69 (5.91−7.92)	73.01 (4.60−165.20)	0.42 (0.33−0.66)
Zhejiang	618.04 (400.15−910.49)	149.63 (108.29−211.12)	6.13 (5.42−7.29)	98.17 (13.08−220.38)	0.74 (0.64−1.09)
**South**					
Guangdong	570.10 (366.92−853.23)	201.67 (148.81−279.34)	8.34 (7.31−9.86)	54.98 (6.77−125.65)	0.46 (0.38−0.71)
Guangxi	667.15 (398.43−1033.52)	173.21 (131.39−230.01)	5.71 (5.10−6.87)	128.23 (14.55−291.53)	1.03 (0.90−1.53)
Hainan	503.76 (329.66−740.93)	153.61 (111.14−209.70)	5.33 (4.73−6.44)	76.66 (9.47−178.41)	0.69 (0.60−1.08)
**Central**					
Henan	641.10 (358.95−1011.60)	205.10 (149.51−273.87)	7.21 (6.23−8.19)	151.15 (19.24−345.54)	1.26 (1.00−1.64)

Parameters	All causes	Alcohol use disorders		Stroke	
	DALYs (/100,000, 95% UI)	DALYs (/100,000, 95% UI)	Indirect economic burden (‱ of GDP)	DALYs (/100,000, 95% UI)	Indirect economic burden (‱ of GDP)
Hubei	666.48 (363.56−1039.59)	177.03 (131.17−241.61)	7.06 (6.31−8.30)	140.28 (13.07−313.07)	1.08 (0.94−1.58)
Hunan	587.81 (356.97−907.38)	148.70 (111.23−196.62)	5.16 (4.64−6.17)	113.59 (14.11−273.18)	0.87 (0.74−1.28)
**Northwest**					
Gansu	609.21 (390.22−946.64)	234.87 (175.94−311.74)	7.35 (6.52−8.84)	99.17 (7.55−235.07)	0.68 (0.58−1.03)
Ningxia	475.01 (321.70−680.66)	198.85 (144.41−265.51)	7.28 (6.52−8.65)	58.75 (6.26−127.25)	0.48 (0.43−0.74)
Qinghai	720.90 (488.33−1069.98)	331.51 (267.73−417.84)	9.03 (7.86−10.88)	88.23 (0.33−241.22)	0.58 (0.49−0.91)
Shaanxi	638.26 (370.79−1016.70)	198.42 (149.30−268.27)	6.49 (5.66−7.78)	129.19 (15.63−291.94)	0.89 (0.76−1.32)
Xinjiang	535.13 (335.48−835.55)	201.44 (146.04−275.96)	7.42 (6.53−8.72)	69.16 (2.71−180.12)	0.59 (0.50−0.89)
**Southwest**					
Chongqing	847.10 (489.27−1388.27)	199.32 (152.63−262.12)	7.49 (6.69−8.85)	123.36 (9.45−285.03)	1.00 (0.87−1.48)
Guizhou	839.61 (566.53−1216.85)	325.68 (243.71−423.67)	9.03 (7.86−11.04)	123.42 (12.82−291.02)	1.00 (0.87−1.52)
Sichuan	899.39 (466.16−1575.70)	185.20 (141.15−246.15)	7.01 (6.24−8.29)	138.11 (7.07−361.35)	1.15 (1.00−1.71)
Xizang	809.38 (492.96−1318.74)	204.72 (150.78−277.58)	6.10 (5.36−7.43)	85.39 (0.10−259.47)	0.50 (0.43−0.84)

Parameters	All causes	Alcohol use disorders		Stroke	
	**DALYs (/100,000, 95% UI)**	**DALYs (/100,000, 95% UI)**	**Indirect economic burden (‰₀ of GDP)**	**DALYs (/100,000, 95% UI)**	**Indirect economic burden (‰₀ of GDP)**
Yunnan	989.76 (692.10−1383.22)	452.56 (350.83−570.69)	15.52 (13.77−18.24)	120.65 (10.68−282.83)	1.10 (0.96−1.65)
**SARs**					
Hong Kong	475.83 (328.85−701.00)	86.66 (60.89−123.29)	3.30 (2.92−3.97)	46.42 (5.86−107.50)	0.46 (0.40−0.68)
Macao	533.80 (359.87−767.14)	168.72 (119.45−231.30)	6.70 (5.89−7.86)	37.31 (3.94−92.13)	0.40 (0.36−0.59)

This table reports provincial and national high alcohol use–attributable deaths and DALY rate (all age) and the corresponding indirect economic burden (‱ of GDP) for 2023, with 95% uncertainty intervals (UIs). DALYs. Disability-adjusted life years; GDP. Gross domestic product; SARs. Special administrative regions

Table S8 All-age DALY rate and indirect economic burden attributable to high alcohol use in China in 2023 (4 diseases)

Parameters	Esophageal cancer		Cirrhosis and other chronic liver diseases		Falls		Liver cancer	
	DALYs (/100,000, 95% UI)	Indirect economic burden (‱ of GDP)	DALYs (/100,000, 95% UI)	Indirect economic burden (‱ of GDP)	DALYs (/100,000, 95% UI)	Indirect economic burden (‱ of GDP)	DALYs (/100,000, 95% UI)	Indirect economic burden (‱ of GDP)
**China**	101.69 (−3.82 to 230.96)	0.79 (0.63−1.17)	72.27 (25.83−124.43)	0.86 (0.66−1.27)	47.67 (20.50−81.34)	1.76 (1.59−2.14)	36.51 (16.31−76.63)	0.38 (0.29−0.55)
**Northeast**								
Heilongjiang	114.03 (−4.34 to 251.96)	0.65 (0.52−1.02)	102.39 (34.63−178.32)	0.87 (0.67−1.26)	30.23 (13.38−53.96)	0.88 (0.78−1.08)	97.21 (42.99−214.07)	0.65 (0.52−0.99)
Jilin	72.31 (−2.59 to 180.53)	0.48 (0.39−0.73)	101.71 (36.18−178.13)	0.95 (0.72−1.42)	22.25 (8.92−40.44)	0.68 (0.61−0.85)	69.76 (33.45−149.85)	0.53 (0.43−0.79)
Liaoning	137.11 (−4.62 to 312.88)	0.86 (0.67−1.31)	110.72 (37.13−194.91)	1.05 (0.80−1.56)	48.19 (20.60−87.44)	1.56 (1.38−1.92)	28.93 (11.70−67.19)	0.22 (0.17−0.33)
**North**								
Beijing	61.65 (−2.59 to 134.99)	0.33 (0.24−0.53)	56.53 (20.37−98.17)	0.50 (0.37−0.76)	66.30 (26.98−117.32)	2.35 (2.08−2.87)	12.47 (5.78−25.83)	0.08 (0.06−0.13)
Hebei	133.65 (−5.51 to 313.48)	0.90 (0.74−1.33)	58.00 (19.89−107.34)	0.61 (0.48−0.90)	39.71 (17.12−73.44)	1.53 (1.40−1.87)	39.07 (19.32−80.45)	0.33 (0.27−0.50)
Inner Mongolia	151.68 (−5.48 to 361.76)	0.97 (0.78−1.47)	88.53 (32.28−153.94)	0.86 (0.66−1.27)	42.93 (18.27−77.30)	1.46 (1.30−1.78)	41.87 (19.54−81.63)	0.32 (0.25−0.48)
Shanxi	90.53 (−2.91 to 218.91)	0.60 (0.49−0.95)	44.09 (15.30−77.80)	0.48 (0.36−0.72)	32.05 (13.01−56.95)	1.05 (0.94−1.28)	22.28 (12.43−43.48)	0.19 (0.15−0.29)
Tianjin	50.00 (−2.34 to 117.78)	0.23 (0.17−0.38)	49.92 (18.34−90.23)	0.41 (0.31−0.63)	31.38 (12.75−56.19)	0.91 (0.81−1.13)	17.79 (8.90−36.66)	0.11 (0.09−0.18)

Parameters	Esophageal cancer		Cirrhosis and other chronic liver diseases		Falls		Liver cancer	
	**DALYs (/100,000, 95% UI)**	**Indirect economic burden (‱ of GDP)**	**DALYs (/100,000, 95% UI)**	**Indirect economic burden (‱ of GDP)**	**DALYs (/100,000, 95% UI)**	**Indirect economic burden (‱ of GDP)**	**DALYs (/100,000, 95% UI)**	**Indirect economic burden (‱ of GDP)**
**East**								
Anhui	106.11<br>(−3.76 to 238.90)	0.87<br>(0.68−1.18)	39.16<br>(13.01−69.54)	0.51<br>(0.38−0.72)	31.02<br>(12.88−52.37)	1.08<br>(0.95−1.27)	23.76<br>(9.23−53.43)	0.28<br>(0.21−0.39)
Fujian	140.38<br>(−5.68 to 305.41)	1.43<br>(1.16−2.19)	61.38<br>(21.24−105.71)	0.85<br>(0.65−1.28)	76.56<br>(35.66−130.20)	3.04<br>(2.73−3.65)	12.21<br>(5.73−26.07)	0.16<br>(0.12−0.25)
Jiangsu	164.98<br>(−6.44 to 390.84)	1.19<br>(0.93−1.85)	38.94<br>(13.15−70.27)	0.47<br>(0.36−0.71)	46.35<br>(18.93−81.48)	1.65<br>(1.48−2.00)	31.43<br>(12.14−68.45)	0.34<br>(0.27−0.54)
Jiangxi	44.79<br>(−1.61 to 110.40)	0.40<br>(0.33−0.59)	86.35<br>(30.21−149.36)	1.10<br>(0.86−1.62)	62.12<br>(28.60−107.88)	2.53<br>(2.30−3.07)	42.53<br>(18.40−86.72)	0.50<br>(0.40−0.75)
Shandong	136.97<br>(−5.69 to 301.75)	1.11<br>(0.88−1.67)	43.02<br>(15.13−76.60)	0.54<br>(0.41−0.77)	33.23<br>(14.19−58.69)	1.21<br>(1.08−1.48)	35.54<br>(16.33−78.71)	0.36<br>(0.29−0.54)
Shanghai	51.98<br>(−2.03 to 121.90)	0.26<br>(0.18−0.43)	38.57<br>(13.96−67.64)	0.33<br>(0.25−0.51)	70.00<br>(28.72−125.73)	2.65<br>(2.35−3.20)	7.06<br>(1.72−16.25)	0.05<br>(0.04−0.08)
Zhejiang	88.64<br>(−3.51 to 194.73)	0.75<br>(0.60−1.14)	59.69<br>(20.31−102.21)	0.72<br>(0.55−1.07)	61.47<br>(26.85−105.07)	2.39<br>(2.14−2.88)	13.75<br>(5.68−28.34)	0.15<br>(0.12−0.23)
**South**								
Guangdong	70.48<br>(−2.32 to 158.69)	0.60<br>(0.46−0.96)	53.33<br>(18.06−94.75)	0.67<br>(0.51−1.02)	54.61<br>(23.95−93.34)	2.21<br>(1.98−2.66)	46.82<br>(18.34−103.52)	0.51<br>(0.40−0.82)
Guangxi	49.50<br>(−2.04 to 111.68)	0.48<br>(0.40−0.75)	122.52<br>(37.63−212.09)	1.54<br>(1.19−2.30)	31.82<br>(13.86−53.88)	1.06<br>(0.96−1.30)	51.19<br>(12.74−122.19)	0.64<br>(0.52−1.03)
Hainan	44.04<br>(−1.62 to 105.68)	0.43<br>(0.34−0.69)	85.22<br>(30.18−146.09)	1.11<br>(0.83−1.72)	32.82<br>(14.49−57.36)	1.17<br>(1.05−1.45)	12.68<br>(4.69−28.79)	0.15<br>(0.12−0.24)

Parameters	Esophageal cancer		Cirrhosis and other chronic liver diseases		Falls		Liver cancer	
	DALYs (/100,000, 95% UI)	Indirect economic burden (‱ of GDP)	DALYs (/100,000, 95% UI)	Indirect economic burden (‱ of GDP)	DALYs (/100,000, 95% UI)	Indirect economic burden (‱ of GDP)	DALYs (/100,000, 95% UI)	Indirect economic burden (‱ of GDP)
**Central**								
Henan	124.95 (−4.48 to 291.48)	0.95 (0.76−1.28)	40.82 (12.99−70.82)	0.52 (0.39−0.72)	45.27 (19.37−77.51)	1.70 (1.49−1.99)	30.31 (14.17−63.75)	0.32 (0.25−0.45)
Hubei	93.82 (−3.65 to 211.67)	0.73 (0.60−1.09)	54.36 (18.05−95.07)	0.61 (0.49−0.89)	61.33 (25.26−106.73)	2.37 (2.15−2.89)	46.30 (18.60−99.25)	0.48 (0.39−0.73)
Hunan	44.24 (−1.41 to 108.60)	0.37 (0.30−0.56)	81.58 (27.33−152.73)	0.91 (0.69−1.35)	29.94 (11.38−52.72)	1.01 (0.91−1.25)	46.99 (20.83−108.52)	0.46 (0.37−0.72)
**Northwest**								
Gansu	65.25 (−2.00 to 166.13)	0.43 (0.34−0.69)	78.67 (27.71−142.29)	0.82 (0.62−1.21)	22.46 (9.45−39.20)	0.69 (0.61−0.84)	23.41 (11.36−49.22)	0.20 (0.16−0.31)
Ningxia	41.73 (−1.48 to 101.84)	0.33 (0.26−0.52)	54.58 (22.11−94.43)	0.69 (0.53−1.05)	45.68 (18.85−78.75)	1.74 (1.56−2.10)	21.90 (9.74−44.94)	0.23 (0.18−0.36)
Qinghai	59.07 (−2.02 to 150.20)	0.37 (0.29−0.59)	95.37 (34.71−177.91)	0.91 (0.67−1.39)	23.62 (10.88−39.70)	0.71 (0.63−0.86)	27.07 (11.69−57.89)	0.22 (0.17−0.34)
Shaanxi	89.54 (−2.87 to 228.72)	0.58 (0.47−0.92)	74.77 (26.68−134.56)	0.81 (0.61−1.21)	49.05 (20.26−83.79)	1.62 (1.45−1.98)	32.66 (16.04−67.98)	0.29 (0.23−0.43)
Xinjiang	76.70 (−2.96 to 205.57)	0.60 (0.49−0.95)	50.15 (16.30−92.74)	0.59 (0.45−0.87)	17.34 (6.94−30.30)	0.64 (0.58−0.78)	14.51 (6.22−31.95)	0.14 (0.11−0.21)
**Southwest**								
Chongqing	164.57 (−6.10 to 400.58)	1.31 (1.05−2.02)	127.87 (85.86−203.79)	1.49 (1.12−2.19)	43.91 (17.78−77.08)	1.57 (1.42−1.92)	65.06 (26.19−141.50)	0.74 (0.59−1.15)

Parameters	Esophageal cancer		Cirrhosis and other chronic liver diseases		Falls		Liver cancer	
	**DALYs (/100,000, 95% UI)**	**Indirect economic burden (‱ of GDP)**	**DALYs (/100,000, 95% UI)**	**Indirect economic burden (‱ of GDP)**	**DALYs (/100,000, 95% UI)**	**Indirect economic burden (‱ of GDP)**	**DALYs (/100,000, 95% UI)**	**Indirect economic burden (‱ of GDP)**
Guizhou	32.00 (−1.10 to 79.11)	0.29 (0.24−0.46)	136.06 (44.86−260.17)	1.67 (1.26−2.61)	59.90 (25.89−102.71)	2.09 (1.90−2.56)	24.19 (9.72−54.68)	0.27 (0.21−0.41)
Sichuan	195.98 (−7.30 to 478.61)	1.75 (1.41−2.69)	118.78 (40.10−207.61)	1.55 (1.20−2.28)	74.64 (32.24−127.37)	3.06 (2.77−3.72)	51.07 (22.54−111.45)	0.61 (0.49−0.94)
Xizang	25.31 (−0.84 to 63.04)	0.16 (0.12−0.25)	266.42 (96.29−537.87)	2.32 (1.74−3.59)	43.54 (17.27−70.83)	1.28 (1.14−1.58)	33.43 (14.54−72.24)	0.25 (0.20−0.39)
Yunnan	42.76 (−1.33 to 102.15)	0.42 (0.34−0.64)	147.02 (48.76−262.45)	1.99 (1.53−2.97)	71.15 (31.17−118.04)	2.92 (2.60−3.50)	34.21 (14.51−73.63)	0.40 (0.31−0.58)
**SARs**								
Hong Kong	36.69 (−1.58 to 84.30)	0.29 (0.23−0.45)	43.72 (15.99−76.54)	0.45 (0.35−0.65)	35.78 (13.71−66.43)	1.30 (1.16−1.59)	53.74 (21.78−118.01)	0.45 (0.35−0.67)
Macao	68.92 (−2.84 to 164.67)	0.60 (0.47−0.92)	26.58 (9.78−54.02)	0.31 (0.24−0.46)	45.78 (19.53−78.64)	1.79 (1.59−2.16)	34.45 (12.48−80.69)	0.38 (0.30−0.59)

This table reports provincial and national high alcohol use–attributable deaths and DALY rate (all age) and the corresponding indirect economic burden (‱ of GDP) for 2023, with 95% uncertainty intervals (UIs). DALYs. Disability-adjusted life years; GDP. Gross domestic product; SARs. Special administrative regions

Table S9 Differences in changes in disease burden and indirect economic burden attributable to high alcohol use in China in 1990

Parameter	All causes	Alcohol use disorders	Stroke	Esophageal cancer	Cirrhosis and other chronic liver diseases	Falls	Liver cancer
**China**	0	0	0	0	0	0	0
**Northeast**							
Heilongjiang	1	0	1	1	1	0	1
Jilin	0	0	1	1	1	0	1
Liaoning	0	1	0	0	0	0	0
**North**							
Beijing	0	0	1	1	1	0	0
Hebei	1	0	0	0	0	0	0
Inner Mongolia	−1	0	0	1	0	0	0
Shanxi	0	0	0	0	0	0	1
Tianjin	0	0	2	1	0	0	1
**East**							
Anhui	0	0	0	0	0	0	0
Fujian	0	0	0	0	0	0	−1
Jiangsu	0	−1	−1	−1	−1	0	0
Jiangxi	0	0	0	0	0	-1	0
Shandong	0	0	0	−1	0	0	0
Shanghai	0	−1	0	1	1	-1	1
Zhejiang	0	0	0	0	0	0	0
**South**							
Guangdong	0	0	0	0	0	0	0
Guangxi	0	0	0	−1	0	1	−1
Hainan	0	0	−1	0	0	0	0
**Central**							
Henan	0	0	0	0	0	0	0
Hubei	0	0	0	0	0	0	0
Hunan	0	0	0	0	0	0	0
**Northwest**							

Parameter	All causes	Alcohol use disorders	Stroke	Esophageal cancer	Cirrhosis and other chronic liver diseases	Falls	Liver cancer
Gansu	0	1	0	0	0	0	0
Ningxia	−1	0	0	0	0	0	0
Qinghai	0	1	0	0	0	0	0
Shaanxi	0	0	0	1	1	1	0
Xinjiang	0	0	0	0	0	1	0
**Southwest**							
Chongqing	2	2	1	2	2	2	2
Guizhou	0	2	0	0	0	0	0
Sichuan	−1	−1	−1	0	−1	−1	−1
Xizang	1	1	0	0	0	0	1
Yunnan	−2	0	0	0	0	−1	0
**SARs**							
Hong Kong	−1	0	−1	0	0	0	0
Macao	0	−1	−1	−1	0	−1	−1

This table quantifies the mismatch between the relative provincial disease burden and relative provincial indirect economic burden in 1990 using a categorical ranking approach (each burden ranked on a 1−5 scale relative to the national distribution). Values represent the difference in ranks (disease rank, indirect economic rank): positive values indicate relatively higher disease burden than indirect economic burden, negative values indicate relatively higher indirect economic burden than disease burden, and 0 indicates concordance. Larger absolute values indicate greater discordance between the two burden dimensions. SARs. Special administrative regions

Table S10 Differences in changes in disease burden and indirect economic burden attributable to high alcohol use in China in 2023

Parameter	All causes	Alcohol use disorders	Stroke	Esophageal cancer	Cirrhosis and other chronic liver diseases	Falls	Liver cancer
**China**	0	0	0	0	0	0	0
**Northeast**							
Heilongjiang	1	0	0	0	1	1	0
Jilin	0	0	1	0	1	0	1
Liaoning	1	0	0	1	1	0	0
**North**							
Beijing	−1	−1	1	1	0	0	0
Hebei	1	0	1	1	1	0	0
Inner Mongolia	0	0	0	0	1	0	0
Shanxi	0	0	0	1	0	0	0
Tianjin	1	1	2	0	1	0	0
**East**							
Anhui	0	0	0	0	0	0	0
Fujian	−1	0	0	−1	0	0	0
Jiangsu	0	0	0	0	0	0	0
Jiangxi	0	0	0	−1	−1	0	−1
Shandong	0	0	0	0	0	0	0
Shanghai	−1	0	1	1	1	−1	0
Zhejiang	0	−1	0	0	0	0	0
**South**							
Guangdong	0	−1	0	0	0	−1	0
Guangxi	0	0	0	−1	0	0	−1
Hainan	−1	1	0	−1	−1	0	0
**Central**							
Henan	0	0	1	0	0	0	0
Hubei	0	0	0	0	0	0	0
Hunan	0	0	0	0	0	0	0
**Northwest**							

Parameter	All causes	Alcohol use disorders	Stroke	Esophageal cancer	Cirrhosis and other chronic liver diseases	Falls	Liver cancer
Gansu	0	1	0	0	0	0	0
Ningxia	−1	0	0	0	0	0	0
Qinghai	0	1	0	1	1	0	0
Shaanxi	0	0	0	1	0	0	1
Xinjiang	−1	0	0	0	0	0	0
**Southwest**							
Chongqing	1	0	0	0	0	0	0
Guizhou	0	1	0	0	0	1	0
Sichuan	0	0	0	0	0	0	−1
Xizang	1	0	0	0	0	1	1
Yunnan	−1	0	0	−1	0	−1	0
**SARs**							
Hong Kong	0	0	0	0	0	0	0
Macao	−1	0	0	0	0	0	0

This table applies the same mismatch metric as **Additional file 1: Table S9** for 1990. Values represent (disease rank-indirect economic rank) on 1−5 relative scales, where positive values indicate provinces with relatively higher disease burden, negative values indicate provinces with relatively higher indirect economic burden, and 0 indicates similar relative levels. Larger absolute values indicate greater discordance between the two burden dimensions. SARs. Special administrative regions

Table S11 Age-specific DALYs and indirect economic burden attributable to high alcohol use in China (male)

Age group (years)	1990		2023	
	DALYs (thousand, 95% UI)	Indirect economic burden (USD, 95% UI)	DALYs (thousand, 95% UI)	Indirect economic burden (95% UI, USD)
<5	0.74 (0.42−1.24)	0.00 (0.00−0.00)	0.35 (0.11−0.65)	0.00 (0.00−0.00)
5−9	14.25 (5.77−27.08)	745,143.91 (271,975.28−1,725,810.49)	11.48 (4.97−22.07)	28,330,064.43 (11,256,965.75−72,299,319.47)
10−14	22.68 (10.90−38.43)	1,091,760.59 (458,532.85−2,264,681.41)	16.40 (8.74−26.45)	36,225,234.80 (15,511,278.85−75,072,492.89)
15−19	180.93 (130.65−245.50)	9,768,787.94 (6,189,077.83−15,867,450.24)	72.33 (51.34−98.08)	151,909,543.57 (98,629,897.61−243,604,877.37)
20−24	660.41 (484.66−877.30)	58,734,092.54 (45,239,336.36−78,924,856.66)	188.37 (135.00−256.49)	762,752,309.00 (610,258,117.93−981,360,801.78)
25−29	795.34 (596.85−1016.00)	83,375,147.81 (65,567,916.63−103,457,420.38)	330.88 (246.01−434.05)	1,655,997,585.58 (1,329,979,286.48−2,020,994,671.82)
30−34	714.02 (526.17−913.14)	75,118,714.00 (60,858,616.81−92,563,093.32)	501.54 (356.49−676.36)	2,583,230,458.10 (2,092,147,737.95−3,120,903,861.62)
35−39	823.67 (579.88−1110.12)	80,989,847.62 (65,663,400.39−97,819,156.00)	647.2 (440.90−883.97)	3,108,325,413.95 (2,477,704,331.38−3,632,046,288.89)
40−44	789.24 (488.97−1147.19)	65,205,893.11 (51,205,494.58−78,484,637.82)	621.93 (389.49−877.93)	2,608,178,260.90 (2,059,711,753.80−3,117,653,240.92)
45−49	810.84 (435.78−1222.41)	52,643,111.84 (40,973,907.32−60,935,912.97)	823.97 (453.65−1251.95)	2,841,474,048.23 (2,280,022,649.93−3,446,633,999.85)

Age group (years)	1990		2023	
	DALYs (thousand, 95% UI)	Indirect economic burden (USD, 95% UI)	DALYs (thousand, 95% UI)	Indirect economic burden (95% UI, USD)
50−54	915.16 (430.68−1539.31)	42,676,018.92 (32,196,197.96−50,542,855.48)	1087.65 (563.87−1727.30)	2,875,079,908.62 (2,198,618,182.53−3,414,712,745.99)
55−59	1027.97 (432.18−1845.16)	31,280,511.38 (22,160,586.38−38,182,232.94)	1140.12 (557.53−1984.06)	2,245,295,779.31 (1,711,709,361.38−2,694,790,882.50)
60−64	1000.17 (405.65−1839.57)	18,247,110.81 (12,983,827.67−22,653,002.46)	924.48 (390.64−1637.11)	1,179,607,246.76 (840,483,879.33−1,456,889,214.33)
65−69	850.09 (348.57−1565.28)	10,774,675.35 (7,223,048.40−13,693,984.07)	907.39 (406.77−1646.10)	891,308,231.39 (624,742,777.67−1,155,823,550.66)
70−74	599.61 (234.45−1113.61)	4,899,173.42 (3,196,837.65−6,620,699.94)	739.72 (330.36−1418.87)	501,614,110.45 (341,733,187.53−696,333,705.34)
75−79	330.88 (140.78−634.22)	1,651,108.39 (973,186.67−2,491,237.62)	459.62 (201.63−915.15)	190,347,521.55 (108,348,024.79−283,194,662.91)
80−84	114.48 (49.27−209.24)	376,424.63 (168,539.37−589,488.77)	239.43 (107.95−492.89)	65,234,633.10 (30,558,345.26−106,287,901.34)
85−89	32.73 (13.37−63.13)	89,115.33 (35,504.35−139,571.27)	124.75 (44.70−265.69)	28,232,015.76 (11,737,367.08−42,327,241.68)
90−94	4.78 (2.07−9.51)	10,145.46 (5012.69−16,543.33)	42.37 (11.08−87.12)	7,888,397.95 (1,333,655.06−7,993,496.39)
>95	0.55 (0.23−1.00)	1275.09 (724.46−1881.54)	11.32 (1.65−24.11)	2,091,483.26 (303,655.80−2,697,082.90)

This table reports age-specific high alcohol use-attributable DALYs and the corresponding indirect economic burden among males in 1990 and 2023. Results illustrate how health loss and productivity loss are distributed across the life course and how the economic burden concentrates in working-age groups. DALYs. Disability-adjusted life years; USD. United States dollar; UI. Uncertainty interval

Table S12 Age-specific DALYs and indirect economic burden attributable to high alcohol use in China (female)

Age group (years)	1990		2023	
	DALYs (thousand, 95% UI)	Indirect economic burden (USD, 95% UI)	DALYs (thousand, 95% UI)	Indirect economic burden (USD, 95% UI)
<5	0.54 (0.33−0.90)	0.00 (0.00−0.00)	0.29 (0.16−0.49)	0.00 (0.00−0.00)
5−9	1.20 (0.63−2.13)	41,249.48 (14,426.28−94,343.20)	0.88 (0.51−1.34)	1,166,759.72 (404,496.20−3,049,507.58)
10−14	2.64 (1.55−4.36)	62,029.08 (23,597.36−127,929.76)	2.03 (1.21−3.10)	1,879,466.90 (743,094.98−4,423,192.90)
15−19	35.27 (25.34−48.33)	2,055,409.76 (1,300,422.66−3,497,083.21)	11.38 (6.74−17.64)	20,635,767.65 (13,357,943.16−31,906,819.02)
20−24	66.78 (46.39−94.20)	6,817,840.60 (5,258,326.98−8,970,723.76)	23.29 (14.50−36.02)	103,085,378.89 (80,502,182.67−131,880,184.83)
25−29	67.13 (48.41−95.20)	8,690,775.34 (6,985,243.91−11,108,803.15)	35.95 (24.25−52.42)	209,808,508.08 (171,161,424.59−263,276,336.54)
30−34	59.57 (42.72−82.56)	7,718,315.93 (6,035,321.01−9,408,713.49)	53.56 (34.75−78.80)	322,319,508.08 (258,878,542.65−403,335,978.83)
35−39	70.12 (52.13−92.96)	8,377,450.41 (6,742,876.70−10,095,289.20)	58.90 (41.02−82.64)	346,364,194.64 (276,521,684.22−426,299,669.99)
40−44	60.95 (42.04−83.06)	6,285,557.15 (4,954,138.46−7,480,598.34)	47.11 (32.45−65.33)	259,013,320.30 (205,887,511.77−314,934,052.15)
45−49	53.49 (35.76−80.06)	4,561,031.07 (3,722,850.39−5,596,083.80)	54.07 (38.66−74.40)	263,872,798.28 (216,600,778.67−321,974,614.89)

Age group (years)	1990		2023	
	DALYs (thousand, 95% UI)	Indirect economic burden (USD, 95% UI)	DALYs (thousand, 95% UI)	Indirect economic burden (USD, 95% UI)
50−54	63.25 (36.76−102.48)	3,814,621.16 (3,041,922.58−4,677,446.38)	70.44 (48.56−105.54)	270,849,435.35 (226,367,253.11−345,965,178.53)
55−59	63.55 (38.58−110.67)	2,694,822.06 (2,152,271.86−3,310,712.38)	70.31 (49.18−102.52)	211,900,219.23 (174,468,392.45−265,953,645.60)
60−64	77.89 (40.51−148.00)	1,952,700.53 (1,471,266.95−2,535,552.23)	55.46 (35.93−81.77)	107,718,450.81 (84,757,395.52−137,103,787.12)
65−69	61.25 (32.75−108.58)	1,142,266.51 (870,602.02−1,493,128.92)	66.46 (41.38−105.74)	94,915,714.53 (73,652,438.63−129,588,001.58)
70−74	55.61 (29.01−105.31)	664,796.27 (468,956.01−910,747.27)	59.77 (38.62−102.49)	58,255,598.00 (42,307,031.29−85,297,749.81)
75−79	33.45 (17.30−61.72)	253,265.12 (158,211.28−380,770.80)	39.73 (24.38−72.98)	23,888,315.20 (14,619,114.94−36,988,731.37)
80−84	14.85 (7.40−29.21)	82,214.65 (36,620.70−124,087.79)	25.38 (13.64−47.42)	10,682,379.33 (5,073,353.18−18,020,739.50)
85−89	5.40 (2.63−10.17)	26,239.81 (12,016.30−40,628.18)	16.78 (7.48−34.40)	6,393,662.71 (2,878,184.67−10,415,936.40)
90−94	1.10 (0.47−2.14)	4403.22 (1343.90−5697.25)	8.00 (2.87−18.54)	2,540,947.10 (1,036,353.28−4,196,060.30)
>95	0.18 (0.05−0.41)	680.68 (392.90−979.94)	2.90 (0.87−7.08)	796,615.02 (329,865.59−1,407,641.41)

This table presents age-specific high alcohol use-attributable DALYs and the corresponding indirect economic burden among females in 1990 and 2023, with 95% UIs. Together with **Additional file 1: Table S11**, these results support sex comparisons in age patterns of health and productivity losses. DALYs. Disability-adjusted life years; USD. United States dollar; UI. Uncertainty interval

Table S13 Model calibration for forecasting the number of YLDs and deaths attributable to high alcohol use using the BAPC model

Year	GBD 2023 results (number, 95% UI)	BAPC results (number, 95% UI)
**YLDs**		
2016	3,510,803.06 (2,451,249.85−4,951,219.59)	3,510,121.63 (3,014,380.64−4,005,862.62)
2017	3,486,630.81 (2,448,908.62−4,942,003.39)	3,586,654.06 (3,035,942.67−4,137,365.46)
2018	3,444,000.65 (2,423,671.99−4,911,771.88)	3,665,520.78 (3,029,026.55−4,302,015.00)
2019	3,406,285.74 (2,409,277.33−4,886,850.52)	3,711,806.92 (2,964,090.01−4,459,523.82)
2020	3,393,201.28 (2,395,124.16−4,857,300.12)	3,757,297.57 (2,869,778.26−4,644,816.87)
2021	3,416,972.72 (2,421,861.02−4,912,809.62)	3,803,585.68 (2,749,549.91−4,857,621.45)
2022	3,437,564.02 (2,447,226.49−4,930,380.82)	3,852,301.02 (2,606,315.43−5,098,286.62)
2023	3,302,920.12 (2,376,235.56−4,708,679.13)	3,903,063.23 (2,440,600.08−5,365,526.37)
**Deaths**		
2016	243,438.79 (115,895.05−437,984.59)	213,926.44 (177,156.25−250,697.07)
2017	244,624.06 (112,834.39−444,329.33)	209,386.83 (170,609.33−248,164.77)
2018	235,946.99 (105,497.31−418,686.10)	205,053.46 (162,721.64−247,385.71)
2019	221,885.62 (101,083.12−395,594.27)	201,020.36 (153,614.02−248,427.12)
2020	213,851.51 (96,259.24−382,004.20)	197,132.69 (143,319.43−250,946.37)
2021	229,395.94 (99,310.48−412,037.04)	193,331.38 (131,984.14−254,679.03)
2022	247,732.15 (103,351.26−451,074.61)	189,725.99 (119,907.39−259,544.97)
2023	233,975.99 (97,493.67−430,070.21)	186,467.76 (107,310.43−265,625.45)

This table compares observed GBD 2023 estimates (deaths and YLDs) with Bayesian age-period-cohort (BAPC) model estimates for the out-of-sample validation period (2016−2023). Model performance is summarized using RMSE and MAPE [with 95% UIs obtained via bootstrap resampling], indicating predictive accuracy for subsequent projections. In the out-of-sample validation period (2016–2023), the RMSE for YLDs was 347,604.97 (95% UI 240,255.40–452,557.61) and the MAPE was 8.82% (95% UI 5.58–12.55). For deaths, the RMSE was 36,578.24 (95% UI 26,611.30–45,825.04) and the MAPE was 14.54% (95% UI 11.08–18.25). BAPC. Bayesian age-period-cohort; GBD. Global Burden of Disease; MAPE. Mean absolute percentage error; RMSE. Root-mean-square error; YLDs. Years lived with disability

Table S14 Forecasting for disease burden and indirect economic burden (% of GDP) attributable to high alcohol use from 2024 to 2050 [% (95% UI)]

Year	Productivity loss due to deaths	Productivity loss due to morbidity	Indirect economic burden
2024	0.04 (0.03−0.05)	0.09 (0.08−0.11)	0.13 (0.11−0.15)
2025	0.04 (0.03−0.05)	0.09 (0.08−0.10)	0.13 (0.11−0.15)
2026	0.04 (0.03−0.05)	0.09 (0.08−0.10)	0.13 (0.11−0.15)
2027	0.04 (0.03−0.05)	0.09 (0.08−0.10)	0.13 (0.11−0.15)
2028	0.04 (0.03−0.05)	0.09 (0.08−0.10)	0.13 (0.11−0.15)
2029	0.04 (0.03−0.05)	0.09 (0.07−0.10)	0.13 (0.10−0.15)
2030	0.04 (0.03−0.05)	0.09 (0.07−0.10)	0.12 (0.10−0.14)
2031	0.04 (0.03−0.05)	0.08 (0.07−0.10)	0.12 (0.10−0.14)
2032	0.04 (0.03−0.05)	0.08 (0.07−0.10)	0.12 (0.10−0.14)
2033	0.04 (0.03−0.05)	0.08 (0.07−0.10)	0.12 (0.10−0.14)
2034	0.04 (0.02−0.05)	0.08 (0.07−0.10)	0.12 (0.10−0.14)
2035	0.04 (0.02−0.05)	0.08 (0.07−0.09)	0.12 (0.10−0.14)
2036	0.04 (0.03−0.05)	0.08 (0.07−0.10)	0.12 (0.10−0.14)
2037	0.04 (0.02−0.05)	0.08 (0.07−0.10)	0.12 (0.09−0.14)
2038	0.04 (0.03−0.05)	0.08 (0.07−0.10)	0.12 (0.09−0.14)
2039	0.04 (0.02−0.05)	0.08 (0.07−0.10)	0.12 (0.10−0.14)
2040	0.04 (0.03−0.05)	0.08 (0.07−0.10)	0.12 (0.10−0.14)
2041	0.04 (0.03−0.05)	0.08 (0.07−0.10)	0.12 (0.10−0.15)
2042	0.04 (0.03−0.06)	0.08 (0.07−0.10)	0.12 (0.10−0.15)
2043	0.04 (0.03−0.06)	0.08 (0.07−0.10)	0.12 (0.10−0.16)
2044	0.04 (0.03−0.06)	0.08 (0.07−0.10)	0.12 (0.10−0.16)
2045	0.04 (0.03−0.06)	0.08 (0.07−0.10)	0.12 (0.10−0.16)
2046	0.04 (0.03−0.07)	0.09 (0.07−0.11)	0.12 (0.10−0.17)
2047	0.04 (0.03−0.07)	0.09 (0.07−0.11)	0.13 (0.11−0.17)
2048	0.04 (0.03−0.07)	0.09 (0.07−0.11)	0.13 (0.11−0.18)
2049	0.04 (0.04−0.08)	0.09 (0.07−0.12)	0.13 (0.11−0.19)
2050	0.04 (0.04−0.09)	0.09 (0.08−0.12)	0.14 (0.12−0.21)

This table reports projected productivity loss due to premature deaths and morbidity, as well as total indirect economic burden, expressed as a percentage of GDP from 2024 to 2050 [95% uncertainty intervals (UIs)]. Projections were generated using the BAPC framework for deaths and YLDs combined with external GDP inputs to compute future economic burden. BAPC. Bayesian age-period-cohort; YLD. Years lived with disability; GDP. Gross domestic product

Table S15 Forecasting for deaths and YLDs attributable to high alcohol use by age groups in 2030, 2040, and 2050

Age group (years)	2030 (number, 95% UI)	2040 (number, 95% UI)	2050 (number, 95% UI)
**Deaths**			
<5	0.01 (0.01−0.01)	0.01 (0.00−0.01)	0.01 (0.00−0.03)
5−9	53.67 (19.35−129.35)	28.47 (7.88−105.10)	33.11 (7.24−229.20)
10−14	95.33 (34.30−215.94)	42.90 (11.85−149.35)	39.23 (8.60−256.76)
15−19	401.13 (212.15−712.71)	218.66 (88.72−597.94)	157.90 (50.92−814.07)
20−24	1442.18 (733.07−2424.99)	1020.29 (399.25−2594.73)	623.53 (193.86−2997.86)
25−29	2586.96 (1334.92−4211.24)	1977.00 (786.84−4636.28)	1465.25 (462.73−6505.36)
30−34	2989.88 (1440.72−5174.10)	2746.56 (1002.56−6503.17)	2647.02 (768.99−11,664.78)
35−39	4125.43 (1744.38−7946.31)	3947.84 (1225.32−10,157.49)	4106.80 (1015.86−18,741.68)
40−44	6526.54 (2255.33−13,425.93)	5910.72 (1486.67−16,454.59)	7389.50 (1454.50−34,659.25)
45−49	9796.79 (2815.35−21,314.53)	9421.82 (2028.75−28,064.82)	12,271.31 (2003.79−60,125.39)
50−54	14,459.34 (3749.07−33,122.06)	13,583.47 (2610.58−42,557.70)	16,747.67 (2420.41−87,440.44)
55−59	23,030.16 (5996.95−54,539.82)	17,411.83 (3179.21−57,356.93)	22,825.49 (3226.36−126,762.88)
60−64	33,811.03 (8874.27−82,480.31)	25,180.42 (4559.83−86,417.16)	32,345.54 (4485.69−186,953.12)
65−69	34,403.80 (9188.67−83,219.76)	37,665.36 (7133.22−128,750.99)	39,183.23 (5376.08−229,419.43)
70−74	30,896.54 (8504.60−74,971.65)	46,920.50 (9339.09−160,230.85)	48,235.12 (6843.77−285,361.86)
75−79	31,497.59 (8965.97−77,676.51)	41,015.28 (8473.83−140,561.79)	62,209.12 (9415.96−370,990.22)
80−84	18,418.58 (5290.48−46,382.03)	24,396.94 (5171.87−84,682.64)	52,875.25 (8374.20−318,056.14)
>85	14,072.57 (3992.64−36,228.96)	24,400.04 (5093.22−85,475.68)	49,475.36 (7744.32−296,583.00)
**YLDs**			

Age group (years)	2030 (number, 95% UI)	2040 (number, 95% UI)	2050 (number, 95% UI)
<5	462.41 (225.74−789.46)	431.35 (212.58−729.80)	570.70 (315.08−947.59)
5−9	1005.09 (559.10−1540.55)	821.58 (456.38−1248.44)	1084.03 (661.67−1617.43)
10−14	4892.85 (2598.03−8055.43)	3618.96 (1905.72−5907.82)	4196.09 (2391.33−6729.01)
15−19	46,019.55 (24,983.86−71,432.06)	42,814.03 (23,024.90−65,813.64)	42,839.24 (24,656.19−64,711.75)
20−24	136,114.08 (83,131.31−205,916.40)	155,836.79 (93,930.22−233,203.68)	141,022.76 (90,343.31−207,407.91)
25−29	204,171.83 (133,245.49−288,334.20)	197,411.88 (126,032.28−276,608.16)	225,179.82 (152,627.37−309,710.34)
30−34	255,879.46 (167,436.70−370,145.44)	208,885.01 (133,388.03−301,617.89)	293,747.59 (198,419.46−415,871.60)
35−39	340,747.22 (230,318.24−470,041.11)	230,943.72 (154,522.45−319,814.50)	273,823.37 (192,108.91−372,917.57)
40−44	401,372.29 (262,448.56−581,384.39)	264,399.03 (178,061.98−382,146.48)	264,490.24 (186,315.85−378,226.19)
45−49	342,852.64 (232,777.21−491,490.58)	331,244.10 (236,849.28−470,632.82)	275,129.17 (208,732.59−388,971.91)
50−54	289,492.61 (191,632.63−423,442.97)	359,651.63 (247,250.90−520,511.12)	290,449.45 (220,436.46−415,749.39)
55−59	301,857.03 (205,739.94−436,015.45)	285,274.80 (199,884.64−405,914.43)	338,304.57 (267,581.32−472,894.47)
60−64	287,232.02 (189,798.00−424,246.12)	234,474.16 (159,262.64−341,713.40)	358,696.34 (271,211.70−512,675.71)
65−69	216,639.68 (139,806.44−324,171.93)	246,156.01 (162,903.31−365,862.29)	288,236.19 (210,187.46−418,297.30)
70−74	150,767.41 (90,317.50−248,224.74)	229,883.05 (139,927.82−379,950.60)	233,246.64 (156,427.87−377,030.39)
75−79	120,408.00 (67,330.65−205,183.85)	157,017.66 (89,401.89−269,721.35)	222,568.42 (139,294.54−376,408.25)
80−84	56,207.51 (26,141.96−95,854.88)	78,641.96 (37,596.78−134,874.40)	154,096.83 (80,235.01−262,972.54)
>85	30,889.89 (8940.42−58,956.45)	59,103.10 (17,477.82−112,969.50)	108,067.93 (34,878.30−206,388.54)

This table presents age-specific projections of high alcohol use-attributable deaths and YLDs for selected future years (2030, 2040, 2050), with 95% UIs. These results describe how the composition of future health loss shifts across age groups under the assumed continuation of historical age-period-cohort patterns. YLDs. Years lived with disability; UI. Uncertainty interval

Table S16 Forecasting for indirect economic burden (% of GDP) attributable to high alcohol use by age groups in 2030, 2040, and 2050

Age group (years)	2030	2040	2050
**Productivity loss due to deaths**			
<5	0.000 (0.000−0.000)	0.000 (0.000−0.000)	0.000 (0.000−0.000)
5−9	0.000 (0.000−0.000)	0.000 (0.000−0.000)	0.000 (0.000−0.000)
10−14	0.000 (0.000−0.000)	0.000 (0.000−0.000)	0.000 (0.000−0.000)
15−19	0.001 (0.000−0.001)	0.001 (0.000−0.001)	0.001 (0.000−0.001)
20−24	0.002 (0.001−0.003)	0.002 (0.001−0.003)	0.002 (0.001−0.003)
25−29	0.003 (0.002−0.004)	0.003 (0.002−0.004)	0.003 (0.002−0.004)
30−34	0.003 (0.002−0.004)	0.003 (0.002−0.004)	0.003 (0.002−0.004)
35−39	0.003 (0.002−0.005)	0.003 (0.002−0.005)	0.003 (0.002−0.005)
40−44	0.004 (0.002−0.006)	0.004 (0.002−0.006)	0.004 (0.002−0.006)
45−49	0.004 (0.002−0.006)	0.004 (0.002−0.006)	0.004 (0.002−0.006)
50−54	0.005 (0.002−0.007)	0.005 (0.002−0.007)	0.005 (0.002−0.007)
55−59	0.005 (0.002−0.007)	0.005 (0.002−0.007)	0.005 (0.002−0.007)
60−64	0.004 (0.002−0.007)	0.004 (0.002−0.007)	0.004 (0.002−0.007)
65−69	0.003 (0.001−0.004)	0.003 (0.001−0.004)	0.003 (0.001−0.004)
70−74	0.001 (0.001−0.002)	0.001 (0.001−0.002)	0.001 (0.001−0.002)
75−79	0.001 (0.000−0.001)	0.001 (0.000−0.001)	0.001 (0.000−0.001)
80−84	0.000 (0.000−0.000)	0.000 (0.000−0.000)	0.000 (0.000−0.000)
>85	0.000 (0.000−0.000)	0.000 (0.000−0.000)	0.000 (0.000−0.000)
**Productivity loss due to morbidity**			
<5	0.000 (0.000−0.000)	0.000 (0.000−0.000)	0.000 (0.000−0.000)
5−9	0.000 (0.000−0.000)	0.000 (0.000−0.000)	0.000 (0.000−0.000)
10−14	0.000 (0.000−0.000)	0.000 (0.000−0.000)	0.000 (0.000−0.000)
15−19	0.000 (0.000−0.000)	0.000 (0.000−0.000)	0.000 (0.000−0.000)
20−24	0.003 (0.002−0.004)	0.004 (0.003−0.005)	0.004 (0.003−0.005)
25−29	0.007 (0.005−0.008)	0.007 (0.005−0.008)	0.008 (0.006−0.011)
30−34	0.009 (0.007−0.011)	0.008 (0.006−0.010)	0.011 (0.008−0.015)
35−39	0.012 (0.010−0.014)	0.008 (0.006−0.010)	0.011 (0.008−0.014)
40−44	0.014 (0.011−0.017)	0.010 (0.008−0.012)	0.010 (0.008−0.014)
45−49	0.012 (0.010−0.015)	0.012 (0.010−0.015)	0.010 (0.008−0.014)
50−54	0.009 (0.007−0.011)	0.011 (0.009−0.014)	0.010 (0.008−0.013)
55−59	0.008 (0.006−0.009)	0.008 (0.006−0.009)	0.010 (0.008−0.013)
60−64	0.005 (0.004−0.007)	0.004 (0.003−0.006)	0.007 (0.006−0.010)

Age group (years)	2030	2040	2050
65−69	0.003 (0.003−0.004)	0.004 (0.003−0.005)	0.005 (0.004−0.007)
70−74	0.002 (0.001−0.003)	0.003 (0.002−0.004)	0.003 (0.002−0.005)
75−79	0.001 (0.001−0.001)	0.001 (0.001−0.002)	0.002 (0.001−0.003)
80−84	0.000 (0.000−0.001)	0.001 (0.000−0.001)	0.001 (0.001−0.002)
>85	0.000 (0.000−0.000)	0.000 (0.000−0.001)	0.001 (0.000−0.001)
**Indirect economic burden**			
<5	0.000 (0.000−0.000)	0.000 (0.000−0.000)	0.000 (0.000−0.000)
5−9	0.000 (0.000−0.000)	0.000 (0.000−0.000)	0.000 (0.000−0.000)
10−14	0.000 (0.000−0.000)	0.000 (0.000−0.000)	0.000 (0.000−0.000)
15−19	0.001 (0.000−0.002)	0.001 (0.000−0.001)	0.001 (0.000−0.001)
20−24	0.005 (0.003−0.007)	0.005 (0.004−0.008)	0.005 (0.003−0.008)
25−29	0.010 (0.007−0.012)	0.009 (0.006−0.012)	0.010 (0.007−0.016)
30−34	0.012 (0.009−0.015)	0.010 (0.007−0.015)	0.014 (0.010−0.022)
35−39	0.015 (0.011−0.019)	0.012 (0.008−0.016)	0.014 (0.010−0.024)
40−44	0.018 (0.013−0.022)	0.013 (0.010−0.019)	0.015 (0.011−0.027)
45−49	0.016 (0.012−0.021)	0.016 (0.012−0.023)	0.017 (0.011−0.029)
50−54	0.013 (0.009−0.017)	0.016 (0.011−0.022)	0.016 (0.010−0.029)
55−59	0.012 (0.009−0.017)	0.011 (0.008−0.016)	0.015 (0.010−0.027)
60−64	0.010 (0.007−0.013)	0.008 (0.005−0.012)	0.012 (0.008−0.023)
65−69	0.006 (0.004−0.009)	0.007 (0.005−0.011)	0.009 (0.006−0.017)
70−74	0.003 (0.002−0.005)	0.005 (0.003−0.008)	0.006 (0.003−0.011)
75−79	0.002 (0.001−0.003)	0.002 (0.001−0.004)	0.004 (0.002−0.008)
80−84	0.001 (0.000−0.001)	0.001 (0.000−0.001)	0.002 (0.001−0.003)
>85	0.000 (0.000−0.000)	0.000 (0.000−0.001)	0.001 (0.000−0.002)

This table reports age-specific projections of productivity loss due to premature deaths, productivity loss due to morbidity, and total indirect economic burden (all as % of GDP) for 2030, 2040, and 2050 (95% UIs). Estimates translate projected Deaths/YLDs into age-patterned productivity losses, highlighting the concentration of future economic impacts in working-age and older age groups. GDP. Gross domestic product; YLDs. Years lived with disability; UI. Uncertainty interval

References

1 Institute for health metrics and evaluation. GBD Results. 2025. https://vizhub.healthdata.org/gbd-results. Accessed 25 Jan, 2026.

2 Briggs A, Claxton K, Sculpher M. Decision modelling for health economic evaluation. Oxford: Oxford University Press; 2006.

3 National bureau of statistics of China. China Statistical Yearbook 2024. 2024. https://www.stats.gov.cn/sj/ndsj/2024/indexch.htm. Accessed 25 Jan, 2026.

4 National bureau of statistics of China. China Population Census Yearbook 2020. 2020. https://www.stats.gov.cn/sj/pcsj/rkpc/7rp/zk/indexch.htm. Accessed 25 Jan, 2026.

5 Penn World Table version 10.0. Groningen Growth and Development Centre: Faculty of Economics and Business, Groningen. 2025. https://www.rug.nl/ggdc/productivity/pwt/. Accessed 25 Jan, 2026.

6 Bai CE, Qian Z. The factor income distribution in China: 1978–2007. China Econ Rev. 2010;21(4):650-70.

7 Burda MC, Hamermesh DS. Unemployment, market work and household production. Econ Lett. 2010;107(2):131-3.

8 Biddle JE, Hamermesh DS. Income, wages and household production theory. Econ Lett. 2020;192:109188.

9 Fang H, Chen C, Fang Y, He X, Hou Z, Jiang M, et al. A guideline for economic evaluations of vaccines and immunization programs in China. Hum Vaccin Immunother. 2022;18(6):2132802.

10 Drummond M, Sculpher MJ, Torrance GW, O'Brien B, Stoddart GL. Methods for the economic evaluation of health care programmes. Oxford: Oxford University Press; 2023.

11 Zhou D, Yang Y, Zhao Z, Zhou K, Zhang D, Tang W, et al. Air pollution-related disease and economic burden in China, 1990-2050: A modelling study based on Global Burden of Disease. Env Int. 2025;196:109300.

12 Institute for health metrics and evaluation (IHME). Gross domestic product per capita 1960-2050−FGH 2023. 2024. https://ghdx.healthdata.org/node/552770. Accessed 25 Jan, 2026.

13 Institute for health metrics and evaluation (IHME). Global fertility, mortality, migration, and population forecasts 2017−2100. 2024. https://ghdx.healthdata.org/record/ihme-data/global-population-forecasts-2017-2100. Accessed 25 Jan, 2026.
